# Crotonylation of GAPDH regulates human embryonic stem cell endodermal lineage differentiation and metabolic switch

**DOI:** 10.1186/s13287-023-03290-y

**Published:** 2023-04-03

**Authors:** Jingran Zhang, Guang Shi, Junjie Pang, Xing Zhu, Qingcai Feng, Jie Na, Wenbin Ma, Dan Liu, Zhou Songyang

**Affiliations:** 1 MOE Key Laboratory of Gene Function and Regulation, Guangzhou Key Laboratory of Healthy Aging Research and SYSU-BCM Joint Research Center, School of Life Sciences, Sun Yat-sen University, Guangzhou, 510275 China; 2 School of Medicine, Tsinghua University, Beijing, 100084 China; 3 Verna and Marrs Mclean Department of Biochemistry and Molecular Biology, Baylor College of Medicine, One Baylor Plaza, Houston, TX 77030 USA; 4 Sun Yat-Sen Memorial Hospital, Sun Yat-sen University, Guangzhou, 510120 China; 5 Bioland Laboratory, Guangzhou, 510320 China

**Keywords:** Embryonic stem cell, Crotonylation, GAPDH, Endodermal differentiation, Metabolic switch

## Abstract

**Background:**

Post-translational modifications of proteins are crucial to the regulation of their activity and function. As a newly discovered acylation modification, crotonylation of non-histone proteins remains largely unexplored, particularly in human embryonic stem cells (hESCs).

**Methods:**

We investigated the role of crotonylation in hESC differentiation by introduce crotonate into the culture medium of GFP tagged LTR7 primed H9 cell and extended pluripotent stem cell lines. RNA-seq assay was used to determine the hESC transcriptional features. Through morphological changes, qPCR of pluripotent and germ layer-specific gene markers and flow cytometry analysis, we determined that the induced crotonylation resulted in hESC differentiating into the endodermal lineage. We performed targeted metabolomic analysis and seahorse metabolic measurement to investigate the metabolism features after crotonate induction. Then high-resolution tandem mass spectrometry (LC–MS/MS) revealed the target proteins in hESCs. In addition, the role of crotonylated glycolytic enzymes (GAPDH and ENOA) was evaluated by in vitro crotonylation and enzymatic activity assays. Finally, we used knocked-down hESCs by shRNA, wild GAPDH and GAPDH mutants to explore potential role of GAPDH crotonylation in regulating human embryonic stem cell differentiation and metabolic switch.

**Result:**

We found that induced crotonylation in hESCs resulted in hESCs of different pluripotency states differentiating into the endodermal lineage. Increased protein crotonylation in hESCs was accompanied by transcriptomic shifts and decreased glycolysis. Large-scale crotonylation profiling of non-histone proteins revealed that metabolic enzymes were major targets of inducible crotonylation in hESCs. We further discovered GAPDH as a key glycolytic enzyme regulated by crotonylation during endodermal differentiation from hESCs.

**Conclusions:**

Crotonylation of GAPDH decreased its enzymatic activity thereby leading to reduced glycolysis during endodermal differentiation from hESCs.

**Supplementary Information:**

The online version contains supplementary material available at 10.1186/s13287-023-03290-y.

## Introduction

Embryonic stem cells (ESCs) hold great therapeutic potential thanks to their ability to self-renew and differentiate into all cell types [1–3]. Pluripotency maintenance and germ layer-specific differentiation of human ESCs (hESCs) are complex processes that require crosstalk between diverse cellular pathways. During hESC differentiation, down-regulation of stem cell-specific markers and up-regulation of germ layer-specific genes depend on the coordinated action of networks of transcription factors and epigenetic regulators. For instance, histones and other chromatin-binding proteins help establish and maintain the unique chromatin states in hESCs, and post-translational modifications (PTMs) (e.g., methylation and acetylation) of these proteins in turn modulate their activities and functions. Additionally, hESCs also undergo germ layer-specific metabolic changes during differentiation [4–7]. While hESCs in pluripotency states rely largely on glycolysis for ATP production, reduced glycolysis and elevated oxidative phosphorylation have been detected during mesoderm and endoderm differentiation [8–11], whereas high glycolytic flux is maintained during ectoderm differentiation [10]. Moreover, metabolic remodeling appears to be a key step in somatic cell reprogramming, where increased glycolysis can enhance reprogramming efficiency from human fibroblasts to inducible pluripotent stem cells [12–15].

Small metabolic intermediates generated by metabolic enzymes can serve as PTM substrates [16–22]. For example, acetyl-CoA (Ac-CoA) is the cornerstone of carbohydrate metabolism and the substrate of protein acetylation. Histone acetylation plays a critical role in the maintenance of hESC open chromatin structure and pluripotency [23–25]. hESCs require high Ac-CoA levels for maintaining histone acetylation modifications as well as pluripotency and self-renewal capacity [20, 26]. During early differentiation, the rapid decrease in glycolysis and Ac-CoA is associated with reduced histone acetylation [20, 26]. While inhibiting Ac-CoA synthesis can lead to hESC differentiation [20, 26], culturing these cells in the presence of the Ac-CoA precursor acetate can delay cell differentiation and block histone deacetylation in a dose-dependent manner [20]. More recently, crotonylation of histones emerged as another important PTM linked to gene regulation [27–29]. The acetyltransferase p300 was shown to also catalyze histone crotonylation, which could stimulate transcription to a greater degree than histone acetylation [28, 30]. In mouse ESCs (mESCs), crotonylation was found to be enriched on histones and decrotonylation resulted in their differentiation [31, 32]. In hESCs, increased histone crotonylation promoted mesoendoderm differentiation [33]. These studies support the importance of histone crotonylation to stem cell fate regulation and underscore the intimate link between PTM and metabolic pathways.

In tumor cells, non-histone proteins involved in cellular functions such as cell cycle and nucleic acid metabolism can be crotonylated on lysines [32, 34]. However, crotonylation of non-histone proteins remains poorly understood, particularly in cells such as hESCs where changes in metabolism and protein modifications can have profound effects on their pluripotency state and differentiation potential. In this study, we explored how changes in protein crotonylation levels affected the transcriptomic profiles and differentiation induction in hESCs of different pluripotency states. Through targeted metabolomic analysis, we found that increased crotonylation also led to reduced glycolysis and enhanced TCA cycle. Furthermore, we identified glycolytic enzymes such as GAPDH that could be crotonylated via mass spectrometry and provided evidence that GAPDH might be a key player during hESC differentiation when cells become less dependent on glycolysis. These findings demonstrate that crotonylation of glycolytic enzymes may be crucial to metabolic switching and cell fate determination in hESCs.

## Materials and methods

### Embryonic stem cell lines and their maintenance and manipulation

All cell lines were routinely tested for mycoplasma and confirmed negative throughout the study. The H9 hESC line expressing the LTR7-GFP reporter was a gift from Dr. Jichang Wang at Sun Yat-sen University. The cells were cultured with daily media change in chemically-defined mTeSR medium (STEMCELL Technologies) on Matrigel (BD Biosciences)-coated plates under 20% O_2_ and 5% CO_2_ at 37 °C, and passaged every 3–5 days with split ratios of 1:3 to 1:5 by adding 0.5 mM EDTA to 3–5 cell clusters [35]. The mouse ESC line E14 was maintained in Dulbecco’s modified Eagle’s medium supplemented with 15% fetal calf serum (Hyclone, Logan, UT, US), 0.1 mM NEAA, 1 mM sodium pyruvate, 0.1 mM β-mercaptoethanol, 100 U/ml penicillin, 0.1 mg/ml streptomycin, 2 mM L-glutamine, LIF (1000 U/ml, Millipore, Billerica, MA), and 2i (3 µM CHIR99021 and 1 µM PD0325901).

The human extended pluripotency stem (hEPS) cells were derived as described previously [36]. Briefly, single-cell suspensions (in LCDM medium) of the H9 LTR7-GFP hESC line were seeded on mitomycin C-inactivated MEF feeder cells in LCDM medium (~ 2 × 10^5^ cells) with medium change daily. After 6–7 days, cells were passaged at ratios of 1:3 to 1:10 every 3–4 days by digestion with 0.05% Trypsin–EDTA and cultured under 20% O_2_ and 5% CO_2_ at 37℃. The derived hEPS cells were removed from feeder cells based on their adherence to the dish bottom and collected for subsequent experiments.

HA-tagged GAPDH and ENOA were cloned into the pET28a vector. hESCs stably expressing HA-tagged proteins were generated by lentiviral infection. Fluorescence microscopy was done on a Zeiss Imager Z1 microscope. Flow cytometry analysis was performed using Beckman CytoFLEX (C01158) and the CytExpert software (Beckman). For drug treatment, cells were cultured in maintenance media containing the appropriate amount of sodium crotonate (Sigma, 391719), sodium acetate (Sigma, S2889), or 3-BrPA (Sigma, 16490) for the indicated amount of time. LCDM medium (50 ml) contains equal parts DMEM/F12 (Thermo, 11330-032) and Neurobasal (Thermo, 21103049) (24 ml each), 250 μl N2 supplement (Thermo, 17502-048), 500 μl B27 supplement (Thermo, 12587-010), 1 mM glutamine (Thermo, 35050-061), 1% non-essential amino acids (Thermo, 11140-050), 0.1 mM β-mercaptoethanol (Thermo, 21985-023), penicillin–streptomycin (Thermo, 15140-122), 5% KSR (Thermo, A3181502), 10 ng/ml recombinant human LIF (Peprotech, 300-05), 1 μM CHIR99021 (Tocris, 4423), 2 μM (S)-(+)-Dimethindenemaleate (Tocris, 1425), 2 μM Minocycline hydrochloride (Sigma, M9511), 1 μM IWR-endo-1(Selleckchem, S7086), and 2 μM Y-27632 (Selleckchem, S1049).

### In vitro differentiation assays

Cells were seeded onto matrigel-coated plates at a density of 1–2 × 10^4^ cells/cm^2^ and cultured in differentiation media to derive endoderm or ectoderm cells as previously described [37, 38]. For endodermal cells, hESCs were cultured in E8 medium containing 5 ng/ml BMP4 and 25 ng/ml Activin A. CHIR99021 (1 μM) was added for the first 2 days only, and the cells were subsequently maintained with medium change daily. For ectodermal cells, hESCs were cultured in MEF-CM medium (Knock-out DMEM containing 20% KSR, 1 mM glutamine, 1% nonessential amino acids, 0.1 mM β-mercaptoethanol, and 1% penicillin–streptomycin) supplemented with 0.1 μM LDN193189 (Selleckchem, S2618) and 10 μM SB431542 (Selleckchem, S1067) with medium change daily.

### RNA-seq and data analysis

RNA-seq analysis was performed by Berry Genomics (Beijing, China). Briefly, total RNA was extracted using the RNeasy Mini Kit (Qiagen) and enriched for mRNA with Oligo (dT), followed by mRNA fragmentation, cDNA synthesis, and PCR amplification. The Agilent 2100 Bioanaylzer and ABI StepOnePlus real-time PCR system were used to analyze sample libraries, which were sequenced using the Illumina HiSeq2500 system in a mode of 150-bp paired-end read. For differential analysis, clean reads of each sample that mapped onto human genome (hg38) with clean ratios above 95.06% were obtained using Tophat (version 1.3.2) and Cufflinks (V1.1.0). The HTSeq (version 0.6.0) was used to quantify mapped counts of human genes. Sequencing read counts were calculated using StringTie (v1.3.0). The expression levels of different samples were normalized by the Trimmed Mean of M values (TMM) and converted into FPKM. The edgeR package of R was used to analyze differential gene expression between samples. Significantly different genes were defined as those with ratios of FPKM greater than 1.5 and *p* values less than 0.05. Functional annotation was performed using the David bioinformatics resource (https://david.ncifcrf.gov/). The functional annotation term categories of biological process (BP), molecular function (MF) and cellular component (CC) were analyzed.

### Targeted metabolomic analysis using HPIC-MRM-MS

Targeted metabolomic analysis was performed by Shanghai BIOTREE Biological Technology Co., Ltd. (Shanghai, China). Briefly, cell samples (1 × 10^7^ cells) were homogenized in 500 μL precooled Chloroform/MeOH (1/3, v/v) for 4 min at 35 Hz and sonicated for 10 min in ice-water. The entire process was repeated three times before the samples were mixed at 4 °C for 15 min, incubated at − 80 °C for 1 h, and centrifuged at 12,000 rpm for 10 min at 4 °C. The supernatant was dried under gentle nitrogen flow and resuspended in 150 μL ultrapure water for filtration and analysis by high-performance ion chromatogram-multiple reaction monitoring-mass spectrometry/mass spectrometry (HPIC-MS/MS) (Thermo Scientific Dionex ICS-6000 HPIC System). Each target peak was detected and different metabolites were left after relative standard deviation de-noising. The missing values were filled up by the median value. The final dataset containing the information of peak number, sample name, and normalized peak area was imported to the SIMCA16.0.2 software package (Sartorius Stedim Data Analytics AB, Umea, Sweden) for multivariate analysis. Data were scaled and logarithmic transformed to minimize the impact of both noise and high variance of the variables. After these transformations, the unsupervised analysis PCA (principle component analysis) that reduces the dimension of the data was carried out to visualize the distribution and grouping of samples. The 95% confidence interval in the PCA score plot was used as the threshold to identify potential outliers in the dataset. Data acquisition occurred on the mass spectrometer operating in negatively polarity multiple reacting monitoring (MRM) mode. MRM data were acquired using the AB SCIEX Analyst Work Station Software (1.6.3 AB SCIEX) and analyzed using MultiQuant 3.0.3. The ThermoFisher Chromeleon processing of targeted metabolites included automated peak detection and integration using the default method and all data were processed by BIOTREE Biological Technology Co., Ltd. (Shanghai, China).

### Protein crotonylation identification using liquid-chromatography fractionation and high-resolution tandem mass spectrometry (LC–MS/MS) and data analysis

Protein crotonylation identification was performed as previously described with minor modifications [32]. Briefly, to enrich for lysine crotonylated peptides, tryptic peptides dissolved in Binding Buffer (20 mM HEPES, pH 8.0, 150 mM KCl, 1 mM DTT, 10% glycerol, 0.1% NP-40, 100 × Cocktail) were incubated at 4℃ overnight with the pan anti-crotonyllysine antibody (4 μg/4 mg peptides) (PTM biolab, PTM-501) that had been pre-coupled to protein A beads (Invitrogen). The bound peptides were washed four times with Wash Buffer (20 mM HEPES, pH 8.0, 150 mM KCl, 1 mM DTT, 0.1% NP-40, 100 × Cocktail) and twice with ultrapure water before being eluted in buffer containing 0.15% TFA and 20% ACN. The eluted peptides were vacuum-dried, cleaned using C18 Spin Columns (10 ng to 30 μg protein) (Thermo, 89870), re-suspended in ddH_2_O containing 0.1% formic acid, and loaded into a nanoViper C18 (Acclaim PepMap 100, 75 μm × 2 cm) trap column in 4 μL/min aliquots. Following trapping and desalting [in 20 μL volume of 100% solvent A (0.1% formic acid)], the peptides were eluted in 60 min on an analytical column (Acclaim PepMap RSLC, 75 μm × 25 cm C18-2 μm 100 Å) with a gradient of 5–38% solvent B (80% acetonitrile, 0.1% formic acid). Tandem MS data were acquired on a Thermo Fisher Q Exactive mass spectrometer (Thermo Fisher, USA) fitted with a Nano Flex ion source using DDA (data-dependent acquisition) mass spectrum techniques. For full mass spectrometry survey scan, the target value was 3 × 10^6^ and the scan ranged from 350 to 2000 *m*/*z* at a resolution of 70,000 and a maximum injection time of 100 ms.

For protein identification and quantification, the MS/MS data were analyzed using PEAKS Studio 8.5. The local false discovery rate at PSM was 1.0% after searching against Homo sapiens database with a maximum of two missed cleavages. Precursor and fragment mass tolerance were set to 10 ppm and 0.05 Da, respectively. All data were then searched against with the UniProt Human database (88817 sequences) including variable modification of crotonylation (lysine, C_4_H_4_O, + 68.03). Crotonylated proteins were analyzed by Kobas based on the Kyoto Encyclopedia of Genes and Genomes (KEGG) databases.

### Seahorse metabolic measurements

The metabolic flux assay was performed using a Seahorse XF96 analyzer (Agilent). hESCs (3 × 10^4^ cells/well) were seeded in the XF96 microplate pre-coated with Matrigel in growth medium. Growth media was changed to Seahorse XF Base Medium (Agilent, 102353-100) supplemented with 2 mM Glutamine before cells were placed in a non-CO_2_ incubator 1 h before the initiation of the test. Extracellular acidification rates (ECAR) were measured in cells that were treated with glucose (10 mM), oligomycin (1 μM), and 2-DG (50 mM) (Agilent Seahorse XF Glycolysis Stress Test Kit, Agilent, 103020-100) according to the manufacturer’s instructions. Seahorse analysis cells were normalized to protein content (determined via Bradford assay) in each well. Data were processed via the Wave software (Agilent). The glycolytic rate results were calculated before and after glucose injection, and glycolytic capacity results were calculated following oligomycin treatment with the initial background measurements subtracted as described in the manufacturer’s instructions.

### In vitro crotonylation and enzymatic activity assays

In vitro crotonylation assays were performed as described previously [28]. Briefly, 0.5 μg of recombinant GAPDH (Ab82633, Abcam) or ENOA (Ab89248, Abcam) proteins were incubated with 0.5 μg of p300 proteins (Active Motif, 81158) in a 30 μl reaction containing 50 mM Tris pH 8.0, 1 mM TCEP, 0.1 mM EDTA, 2 mM MgCl_2_, and 20 μM Crotonyl-CoA (Sigma, 28007) for 1 h at 30 °C. The reactions mixtures were then used for activity assays using the GAPDH and Enolase Activity Assay Kits (Sigma, MAK277 and MAK178) following the manufacturer’s instructions. Alternatively, cells were lysed and incubated with Assay Buffer before enzymatic activity assays. GAPDH activity was determined based on the generation of NADH per minute at pH 7.2 at 37 °C. Enolase activity was determined based on the amount of enzyme needed to generate 1.0 nmole of H_2_O_2_ per minute at pH 7.2 at 25 °C.

### Immunofluorescence

Immunofluorescence assay was performed according to standard procedures. Cells were fixed with freshly prepared 4% paraformaldehyde in PBS at 4 °C for 15 min, permeabilized in 0.1% Triton X-100 in PBS at room temperature for 30 min, washed three times, and left in blocking solution (4% goat serum in PBS) for 1 h. Samples were incubated with primary antibody dissolved in blocking solution at 4 °C overnight, washed three times and incubated for 1 h with secondary antibodies at room temperature. Samples were washed, and the nuclei were stained with DAPI in Vectashield mounting medium. Fluorescence was detected and imaged using a Zeiss Imager Z1 microscope. The anti-NANOG (ab80892, Abcam), anti-OCT4 (ab181557, Abcam) and anti-GATA6 (5851, Cell signaling technology) antibodies were used at 1:200 dilution. Secondary antibodies including AlexaFluor 488 anti-rabbit IgG (A-21206, 1:1000), and AlexaFluor 555 anti-Mouse IgG (A-31570, 1:1000) were obtained from Invitrogen. Images were observed under the Carl Zeiss Axio Image A2 Microscope and acquired by ZEN 2.3 software. The measured resolution is 1388 × 1040 with 63 × 1.40 oil apochromat.

### Protein immunoprecipitation and western blotting

A total of 1 × 10^7^ cells were lysed in lysis buffer (40 mM Tris pH 8.0, 100 mM NaCl, 1 mM EDTA, 10% glycerol, 0.5% NP40) containing protease inhibitors. Protein concentration was measured using BCA protein assay (Thermo Fisher) and normalized across samples prior to immunoprecipitation. For Kcr immunoprecipitation, 5 μg of antibody and 50 μL of protein A beads were added to lysates overnight at 4 °C. Lysates were centrifuged for 3 min at 4 °C, and the beads were washed three times with ice-cold lysis buffer and resuspended in lysis buffer and boiled for 5 min.

Western blotting was performed according to standard procedures. Cells were resuspended in RIPA Buffer containing 50 mM Tris (pH 7.4), 150 mM NaCl, 1 mM EDTA, 1% Triton X-100, 10% glycerol, 0.25% deoxycholate, and 0.1% SDS supplemented with protease inhibitor cocktail (Sigma) on ice for 20 min. Lysates were resolved by SDS–PAGE and blotted onto polyvinylidene difluoride (PVDF) membranes. Membranes were blocked in 5% nonfat milk/TBST and incubated with primary antibodies at 4 °C overnight. Antibodies used in the study include anti-crotonyllysine (PTM Biolab, PTM-501, 1:1000), anti-β-actin (Abclonal, AC026, 1:5000), anti-histone H3 (Abcam, ab1791, 1:5000), anti-GAPDH (Proteintech, 60,004–1, 1:5000), anti-GAPDH (Millipore, MAB374), anti-HA (Sigma, H9658, 1:5000). Odyssey IRDye 680CW anti-mouse (926–32,220), and IRDye 800CW anti-rabbit (926–32211) secondary antibodies were used at 1:5000 dilution.

### Reverse transcription quantitative real-time PCR (RT-qPCR)

RT-qPCR was performed as previously described. Briefly, total RNA was isolated using the RNeasy Mini Kit (Qiagen) and reversely transcribed using reverse transcriptase (Vazyme, R223). Real-time quantitative PCR reactions were conducted using Power SYBR Green PCR Master Mix (Vazyme, Q321) on an ABI Prism 7300 Sequence Detection System. The data were analyzed by the delta-delta CT method. The expression level of each gene was normalized with actin RNA. Primers are listed in Additional file 2: Table S7.

### Protein extraction for mass spectrometry analysis

Protein extraction was done essentially as described previously. Cells were resuspended in 400 μl of lysis buffer (8 M urea and cocktail protease inhibitors (Sigma), 20 mM HEPES, pH 8.0). The samples were sonicated at 20% power in a tip sonicator and incubated on ice for 20 min. The supernatant was collected after 15 min of centrifugation at 20,000*g* at 4 °C, and the protein concentration was determined by BCA assay (Thermo Fisher). For digestion, 2–4 mg proteins were reduced in 500 mM DTT for 1 h at 60 °C and alkylated with 1 M iodoacetamide at room temperature for 30 min protected from light. For trypsin digestion, the protein samples were diluted by adding 50 mM NH_4_HCO_3_ pH 8.0 to reduce the urea concentration below 2 M. Trypsin was added at 1:100 trypsin-to-protein mass ratio for an overnight digestion. The samples were desalted by C18 Column (Maximum 500 mg protein) (Waters, 186004619) and the peptides were dried by vacuum centrifugation.

### Site-directed mutagenesis and protein purification

The GAPDH single and double lysine mutants were generated using the Quickchange II site-directed mutagenesis kit (Agilent Technologies). pET28a plasmids encoding His-tagged wild-type or mutant GAPDH were expressed in BL21 Escherichia coli. HisSep Ni–NTA Agarose Resin (YEASEN, 20503ES1) was used for protein purification. Cells were cultured at 30 °C for 6 h, harvested, and resuspended in lysis buffer (50 mM NaH2PO4, 300 mM NaCl, 10 mM imidazole, pH8.0). Following sonication and centrifugation, the supernatant was loaded onto a nickel agarose resin column pre-equilibrated with lysis buffer. The column was washed with 5 × volumes of wash buffer (lysis buffer with 20 mM imidazole) and the proteins eluted with elution buffer (lysis buffer with 250 mM imidazole). After purification, proteins were dialyzed at 4 °C overnight.

### GAPDH shRNA knockdown (KD)

DNA oligos encoding the shRNA sequence targeting GAPDH were cloned into the pLV-H1-EF1α-puro vector (Biosettia #SORT-B19). Lentiviruses encoding the shRNAs were ultracentrifuged at 70,000×*g* at 4 °C for 2 h before immediate use or storage at − 80 °C. Reporter hESCs seeded on matrigel-coated plates were infected with the viruses and selected in 2 μg ml^−1^ puromycin for 3 days. The following sequence was used:

$${5}{\prime }{ - } {\text{AAAAGCACCTTGTCATGTACCATTTGGATCCAAATGGTACATGACAAGGTGC}}.$$

### Statistical analysis

Statistical tests were performed by GraphPad Prism. A value of *p* < 0.05 was considered statistically significant.

## Results

### Distinct transcriptomic changes occur in crotonate-treated primed hESCs

**Fig. 1:**
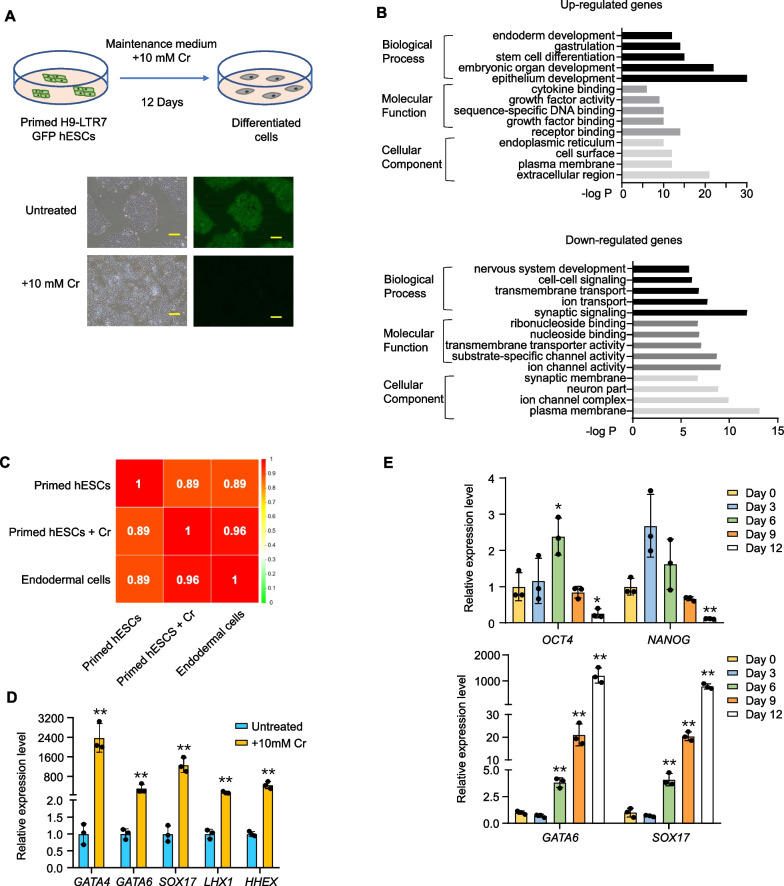

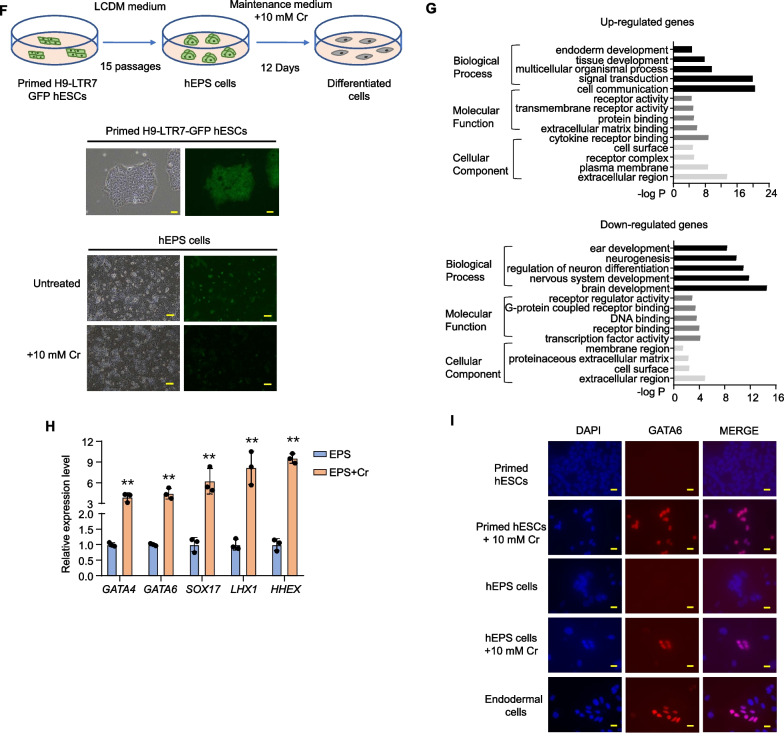
Crotonate-treated hESCs display transcriptomic changes associated with endodermal development. **A** H9-LTR7 GFP reporter hESCs were cultured in crotonate (Cr)-containing (10 mM) maintenance medium for 12 days before phase-contrast and fluorescence microscopy. Scale bar, 100 μm. **B** Cells from **A** were harvested after 12 days of Cr treatment for RNA-seq. Untreated H9-LTR7 GFP reporter hESCs served as controls. Results from gene ontology (GO) analysis of genes up- or down-regulated with treatment were plotted as shown. **C** Results from **B** were compared to RNA-seq data using endodermal cells derived from H9-LTR7 GFP reporter hESCs to calculate the Pearson correlation coefficients. **D** Cells from **A** were harvested after 12 days of Cr treatment for RT-qPCR analysis of the indicated endodermal development genes. Untreated cells served as controls. Error bars represent mean ± S.D. (*n* = 3 independent experiments). Significance was calculated using unpaired *t* test. **p < 0.01. **E** H9-LTR7 GFP reporter hESCs were cultured in crotonate (Cr)-containing (10 mM) maintenance medium and harvested for RT-qPCR analysis at the indicated time points for pluripotency (top) or endoderm (bottom) markers. Error bars represent mean ± S.D. (*n* = 3 independent experiments). Significance was calculated using unpaired *t* test. *p < 0.05, **p < 0.01. **F** H9-LTR7 GFP reporter hESCs were cultured first in LCDM medium for 15 passages to derive hEPS cells, which were then cultured in crotonate (Cr)-containing (10 mM) maintenance medium for 12 days before phase-contrast and fluorescence microscopy. Scale bar, 100 μm. **G** Cells from **F** were harvested after 12 days of Cr treatment for RNA-seq and GO analysis. **H** RT-qPCR analysis for the indicated marker genes. Error bars represent mean ± S.D. (*n* = 3 independent experiments). Significance was calculated using unpaired *t* test. **p < 0.01. **I** H9-LTR7 GFP hESCs (±10 mM Cr for 12 days), converted EPS cells (±10 mM Cr for 12 days), and endodermal cells derived from the reporter hESCs were immunostained with an anti-GATA6 antibody. DAPI was used to stain the nuclei. Scale bars, 10 μm

Fluctuations in the level of intermediate metabolites such as crotonyl-CoA can affect not only the equilibrium between different metabolic pathways but also the degree and type of PTM on proteins important for chromatin remodeling and gene expression [27, 29, 39]. Crotonyl-CoA is generated from lysine/tryptophan metabolism and mitochondrial/peroxisomal fatty acid oxidation [40–45]. Adding the crotonyl-CoA precursor crotonate to cultures can increase the cellular level of crotonyl-CoA as well as protein crotonylation [27, 33]. To better understand the role crotonylation plays in cell fate determination and pluripotency maintenance in hESCs, we took advantage of the H9 hESC line that expresses the LTR7/HERVH-driven GFP transgene [35] and examined their transcriptional profiles following crotonate treatment by RNA-seq. LTR7/HERVH transcription is required for maintaining hESC pluripotency [46], and the expression of pluripotency genes directly correlates with HERVH levels. The GFP reporter therefore enables easy visualization and tracking of pluripotent hESCs during long-term culturing and differentiation induction. As shown before, the vast majority of the reporter cells had relatively low levels of GFP expression and normal expression of pluripotency markers such as NANOG and OCT4 (Fig. 1A, Additional file 1: Figs. S1A-B), indicating that they were in the primed pluripotency state. When we induced the reporter hESCs to differentiate into endodermal (with BMP4 and Activin A, Additional file 1: Fig. S1C) or ectodermal cells (with BMP pathway inhibitors, Additional file 1: Fig. S1D), we could observe GFP signal reduction that was accompanied by increased expression of germ layer-specific markers (Additional file 1: Figs. S1E–H).

We thus cultured the H9-LTR7 GFP reporter hESCs in crotonate-containing (10 mM) maintenance medium for 12 days before harvesting the cells for RNA-seq. While the untreated hESCs were able to maintain GFP expression and the primed pluripotency state morphology, crotonate-treated hESCs gradually lost GFP signals (from 100 to 15%) although the edges of the colonies had not yet rounded up (Fig. 1A), indicating a more differentiated state. Based on GO and KEGG analyses, coinciding with these changes was the up-regulation of genes involved in biological processes such as endoderm development, stem cell differentiation, and embryonic organ development with molecular functions of receptor or growth factor binding (Fig. 1B, Additional file 2: Table S1). Meanwhile, genes implicated in nervous system development and synaptic signaling with functions of transmembrane transporter and ion channel activities were down-regulated (Fig. 1B, Additional file 2: Table S2). When compared with endodermal cells derived from the reporter cells, the Pearson correlation coefficients indicate that the crotonate-treated cells at day 12 resembled more closely the derived endodermal cells (Fig. 1C). These observations indicate that crotonate treatment led to the respective activation and inhibition of endodermal and ectodermal gene expression in hESCs. We next verified our RNA-seq results by RT-qPCR analysis. Indeed, endoderm markers such as *GATA4*, *GATA6*, *SOX17*, *LHX1,* and *HHEX* became highly expressed in crotonate-treated cells (Fig. 1D). These findings are supported by results from the recently published report that also examined crotonate-treated hESCs [31]. A closer look at gene expression during the 2-week treatment period revealed that pluripotency marker genes (e.g., *OCT4* and *NANOG*) initially increased moderately and then decreased to fairly low levels (Fig. 1E, top). Endoderm markers such as *GATA6* and *SOX17* on the other hand exhibited a gradual and steady increase in the presence of crotonate (Fig. 1E, bottom). Taken together, our results suggest that crotonate treatment led to germ layer-specific differentiation of the primed hESCs with a bias towards the endodermal lineage.

### Crotonate-treated hEPS cells exhibit transcriptomic changes similar to primed hESCs

Primed hESCs have been found to transcriptionally resemble the late post-implantation epiblast in non-human primate models [47, 48], and can be converted to extended pluripotent stem (EPS) cells capable of giving rise to both embryonic and extraembryonic tissues in vivo [36, 49, 50]. The ability to obtain EPS cells that are in a more naïve pluripotent state has enabled the investigation of aspects of human development not possible in primed hESCs, for example, the mechanisms of how naive hESCs switch from pre-implantation transcriptional and epigenetic programs to differentiation-competent programs. Given that the various interconvertible pluripotency states are discrete and that EPS cells are distinct from primed hESCs molecularly and functionally, we set out to examine whether EPS cells might respond to crotonate treatment differently from primed hESCs.

When the reporter hESCs were cultured in LCDM medium [36], they became more dome-shaped while maintaining similar levels of GFP signals and pluripotency marker expression (e.g., *NANOG* and *OCT4*) (Fig. 1F, Additional file 1: Figs. S1A–C), indicating their conversion to the more naïve-like EPS cell state as previously reported. We cultured the converted EPS cells in the presence of crotonate and performed RNA-seq analysis as described above. Compared to the primed-state reporter cells, fewer converted hEPS cells lost LTR7-GFP signals (from 100 to 26%) with crotonate treatment (Fig. 1F). RNA-seq results from the treated hEPS cells found a similar enrichment of pathways that were important in endodermal cell differentiation [51–53] in upregulated genes (Fig. 1G, Additional file 2: Table S3). At the same time, downregulated genes were enriched with those involved in ectodermal cell differentiation (ear development and neurogenesis) [54–56] (Fig. 1G, Additional file 2: Table S4). We again verified the RNA-seq results by RT-qPCR analysis (Fig. 1H) as well as immunofluorescence (IF) assays (Fig. 1I). Interestingly, compared to primed hESCs, where the expression of endoderm marker genes went up > 1000 fold (Fig. 1D), crotonate-treated EPS cells exhibited ~ 15-fold increase in endoderm marker gene expression (Fig. 1H). These results suggest that increased cellular crotonyl-CoA levels led to similar global molecular changes in EPS cells and primed hESCs. More careful examination of the subtle differences in their transcriptomic signatures may shed light on the underlying mechanisms that govern the differentiation potential of hESCs of different pluripotency states during development.

### Increased protein crotonylation leads to ESC differentiation into the endodermal lineage

**Fig. 2:**
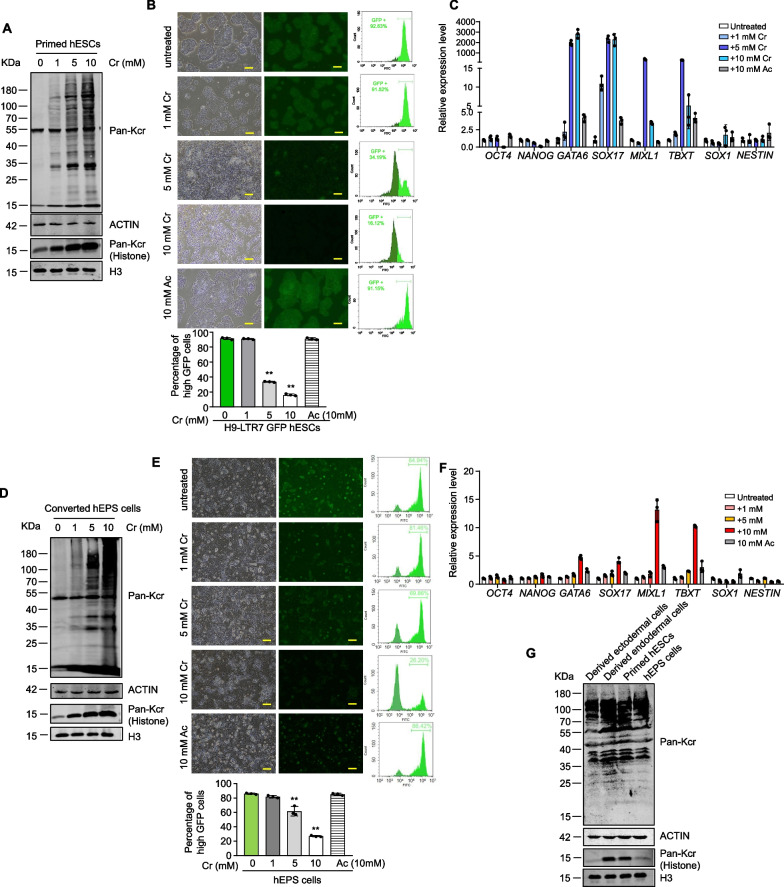
Increased protein crotonylation upon crotonate treatment promotes hESC differentiation into the endodermal lineage. **A** H9-LTR7 GFP hESCs were cultured in different concentrations of crotonate for 24 h before being harvested for western blotting as indicated. **B** H9-LTR7 GFP hESCs were cultured in different concentrations of crotonate for 12 days before analysis by microscopy (left) and flow cytometry (right). The percentage of GFP-expressing cells was plotted below. Cells cultured without crotonate or with 10 mM acetate (Ac) for 12 days served as controls. Scale bar, 100 μm. Error bars represent mean ± S.D. (*n* = 3 independent experiments). Significance was calculated using unpaired *t* test. **p < 0.01. **C** Cells from **B** were analyzed by RT-qPCR for marker gene expression. *OCT4* and *NANOG*, pluripotency markers. *GATA6* and *SOX17*, endoderm markers. *MIXL1* and *TBXT*, mesoderm markers. *SOX1* and *NESTIN*, ectoderm markers. Error bars represent mean ± S.D. (*n* = 3 independent experiments). **D** hEPS cells derived from reporter hESCs were cultured and western blotted as described in **A**. **E**, **F** hEPS cells derived from reporter hESCs were treated as described in **B** and similarly harvested for microscopy and flow cytometry **E** and RT-qPCR analysis **F**. Scale bar, 100 μm. Error bars represent mean ± S.D. (*n* = 3 independent experiments). Significance was calculated using unpaired *t* test. **p < 0.01. **G** H9-LTR7 GFP hESCs, converted EPS cells, and ectoderm and endoderm cells derived from the reporter hESCs were western blotted with the indicated antibodies

Studies of crotonylation have largely focused on histones and the role of histone crotonylation in stimulating transcription. We found that even relatively brief crotonate treatment led to a dose-dependent increase in the crotonylation of both histones and non-histone proteins in primed reporter hESCs (Fig. 2A). The expression of mesoendoderm marker genes (e.g., *GATA6*, *SOX17*, *MIXL1* and *TBXT*) was clearly induced (> 1000 fold) along with significant loss of GFP signals (Figs. 2B, C). Similarly, GFP signal loss was already detectable in the converted reporter hEPS cells with 5 mM crotonate, despite more modest induction of lineage marker expression (Fig. 2D–F). When we examined the level of protein crotonylation using a pan anti-crotonyl-lysine (Kcr) antibody, we saw clearly elevated histones crotonylation in primed hESCs compared to hEPS cells (Fig. 2G). Notably, the level of non-histone crotonylation in endodermal cells differentiated from primed hESCs displayed higher crotonylation compared to primed hESCs, whereas the level of crotonylation in ectodermal cells differentiated from primed hESCs was lower. These results are in line with our RNA-seq data linking increased protein crotonylation with germ layer-specific hESC differentiation. Next, we cultured the mouse ESC line E14 in different concentrations of crotonate and examined its ability to differentiate. Similar to hESCs, mESCs also exhibited a dose-dependent increase in protein crotonylation (Additional file 1: Fig. S2A) that correlated with increased percentages of differentiated clones and elevated expression of mesoendodermal markers (Additional file 1: Figs. S2B–F). Our findings combined indicate that crotonate treatment can induce stem cell differentiation towards endodermal cells and implicate crotonylation in differential cell fate determination.

### Crotonate-treated hESCs show reduced glycolysis and enhanced tricarboxylic acid (TCA) cycle

**Fig. 3:**
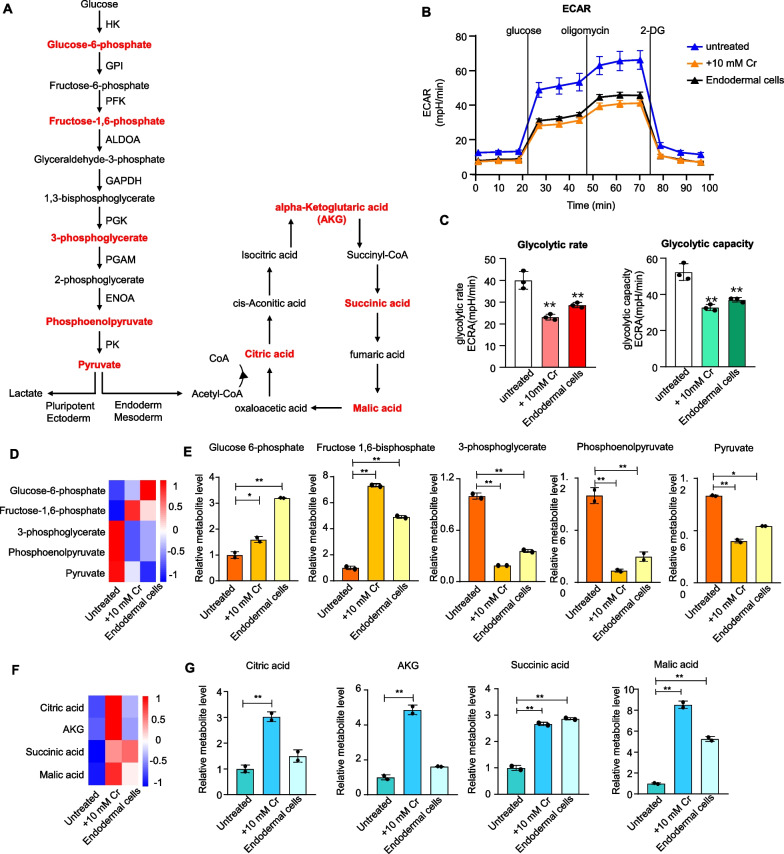
Crotonate treatment leads to reduced glycolysis and enhanced TCA cycle. **A** Key metabolic intermediates in glycolysis and the TCA cycle are shown. Red, intermediates measured by HPIC-MRM-MS/MS. **B** Extracellular acidification rate (ECAR) analysis of untreated H9-LTR7 GFP cells, endodermal cells, and reporter hESCs treated with 10 mM crotonate for 12 days. Glucose (10 mM), oligomycin (1 mM), and 2-deoxyglucose (2-DG, 50 mM) were added as indicated. **C** Glycolytic rates and glycolytic capacity (ECAR following oligomycin) for untreated H9-LTR7 GFP cells, endodermal cells, and reporter hESCs treated with 10 mM crotonate for 12 days. Error bars represent mean ± S.D. (*n* = 3 independent experiments). Significance was calculated using unpaired *t* test. **p < 0.01. **D**, **E** Reporter hESCs were cultured in crotonate-containing media (10 mM) for 12 days before targeted metabolomic analysis. Untreated cells and endodermal cells differentiated from the reporter hESCs were used as controls. Levels of the measured intermediates were normalized to standards. The results for several glycolytic intermediates and the end product pyruvate were compared across samples in a heatmap (**D**) or individually plotted (**E**). Error bars represent mean ± S.D. (*n* = 3 independent experiments). Significance was calculated using unpaired *t *test. *p < 0.05; **p < 0.01. **F**, **G** Based on metabolomic data, results for several TCA cycle intermediates were compared across samples in a heatmap (**F**) or individually plotted (**G**). Error bars represent mean ± S.D. (*n* = 3 independent experiments). Significance was calculated using unpaired *t* test. *p < 0.05; **p < 0.01

During aerobic respiration, glucose is converted through glycolysis into pyruvate, which delivers the acetyl group to the TCA cycle for energy production [57]. The pyruvate dehydrogenase reaction removes the carboxyl group to form acetyl-CoA, thus providing the primary link between glycolysis and the TCA cycle (Fig. 3A). A metabolic switch with decreased glycolysis and elevated oxidative phosphorylation can occur during hESC differentiation into endodermal cells [10, 11]. Indeed, when we performed the seahorse metabolic assays, endodermal cells derived from Activin A treatment exhibited lower extracellular acidification rate (ECAR) as well as lower glycolytic rate and capacity compared with untreated hESCs (Fig. 3B, C). Importantly, coronate-treated reporter hESCs too showed down-regulated ECAR, glycolytic rate, and glycolytic capacity, indicating reduced glycolysis with crotonate treatment.

We next investigated whether such metabolic changes were accompanied by the concurrent increase/decrease of specific intermediate metabolites. To this end, we carried out targeted metabolomic studies by HPIC-MRM-MS/MS to measure the levels of key intermediate metabolites in glycolysis and the TCA cycle. For controls, we again used untreated hESCs as well as endoderm cells derived from the reporter hESCs. Consistent with previous findings, the derived endodermal cells showed an accumulation of upstream glycolytic intermediates (e.g., glucose 6-phosphate and fructose 1,6-bisphosphate) and a corresponding decrease in downstream intermediates (e.g., 3-phosphoglycerate and phosphoenolpyruvate) as well as the end product pyruvate (Fig. 3D, E), suggesting reduced glycolysis. Crotonate treatment in hESCs led to similar shifts in the glycolytic pathway. When metabolites of the TCA cycle were determined, a specific accumulation of alpha-ketoglutaric acid and citric acid was seen with crotonate treatment. Crotonate-treated cells and to a lesser extent the derived endodermal cells exhibited enhanced flux through the TCA cycle relative to untreated hESCs (Fig. 3F, G), indicating more active TCA cycle in these cells. Together, these results show that increased crotonylation and induced differentiation are both associated with a shift from glycolysis to oxidative phosphorylation in crotonate-treated hESCs.

### Large-scale identification of lysine-crotonylated proteins in hESCs

**Fig. 4:**
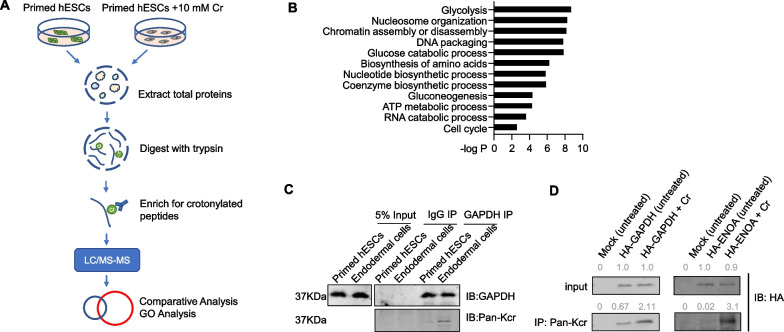
Large-scale analysis of lysine-crotonylated proteins in crotonate-treated hESCs identifies glycolytic enzymes as major targets. **A** Reporter hESCs were cultured in crotonate-containing media (10 mM) for 12 days before being harvested along with untreated hESCs for protein extraction and peptic digest. Crotonylated peptides were enriched with an anti-Kcr antibody before LC/MS–MS analysis. **B** GO analysis of crotonylated proteins in response to crotonate treatment that were identified by LC/MS–MS. **C** H9-LTR7 GFP reporter hESCs and endodermal cells derived from the reporter cells were immunoprecipitated (IP) with an anti-GAPDH antibody. The immuoprecipitates were then western blotted as indicated. IgG served as a negative control. **D** Reporter hESCs stably expressing HA-tagged GAPDH (left) or ENOA (right) were cultured in crotonate-containing media (10 mM) for 24 h before being harvested for IP with the anti-Kcr antibody and western blotting with an anti-HA antibody. Parental (Mock) and untreated cells served as controls

The relative concentration of intermediate metabolites (e.g., acetyl-CoA vs. crotonyl-CoA) directly impacts the rate and extent of protein acylation. Addition of acetate, the acetyl-CoA precursor, could increase glycolysis and global protein acetylation, accompanied by delayed differentiation of primed hESCs [20]. Similarly, adding crotonate to cells could increase global protein crotonylation [28, 32]. In hESCs, drastically increased crotonylation of non-histone proteins was clearly evident upon crotonate treatment (Fig. 2A). We speculated that crotonylation of non-histone proteins must also be crucial to the differentiation induction process, given the markedly changed levels of metabolic intermediates upon crotonate addition. To uncover the identities of such proteins, we performed proteomic analysis by LC–MS/MS and enriched for crotonylated peptides/proteins with the pan anti-Kcr antibody following tryptic digestion (Fig. 4A). In the absence of crotonate, five proteins (TBA1C, FLNA, RYR2, APBP2, and RS24) were found to be crotonylated. That number increased to 53 with crotonate treatment where only TBA1C and APBP2 appeared in both sets (Additional file 2: Table S5). The list also contains known crotonylated proteins such as histone H2B1 and nucleophosmin 1 (NPM1) [32]. It is clear that functionally diverse proteins undergo crotonylation in crotonate-treated primed hESCs, including those involved in nucleosome organization, chromatin assembly or disassembly, and DNA packaging (Fig. 4B).

Of the many candidates in our list, we decided to focus on proteins with enzymatic activities, because small-molecule inhibitors/activators may be available or developed to manipulate their activities. In particular, metabolic enzymes GAPDH and ENOA, which participate in glycolysis and glucose catabolic processes, were highly enriched (Fig. 4B, Additional file 2: Table S6). Epigenetic modifications of GAPDH (acetylation [58, 59] or malonylation [60]) and ENOA (acetylation [58] or crotonylation [61]) have been shown to affect their activities in response to glucose, suggesting that these enzymes may be prime PTM targets for controlling glycolysis. In crotonate-treated cells, crotonylation of metabolic enzymes may thus facilitate the metabolic shifts necessary for hESC differentiation. To explore this idea further, we first immunoprecipitated GAPDH from endodermal cells and untreated reporter hESCs for western blotting with the anti-Kcr antibody. As shown in Fig. 4C, we could readily detect crotonylated GAPDH in endodermal cells but the signal was nearly negligible in untreated hESCs. We reasoned that the difficulty in detecting endogenous GAPDH crotonylation in untreated hESCs might be because the level of endogenous GAPDH crotonylation in these cells was below the detection threshold of the anti-Kcr antibody. Additionally, although we tested several antibodies against ENOA, none worked well in immunoprecipitation assays. We therefore generated reporter hESCs that stably expressed HA-tagged GAPDH or ENOA and immunoprecipitated these tagged proteins using the anti-Kcr antibody. Even without crotonate treatment, the anti-Kcr antibody could bring down both HA-GAPDH and HA-ENOA, whose signals increased further with crotonate treatment (Fig. 4D).

To further investigate GAPDH crotonylation during crotonate-induced hESC differentiation, we immunoprecipitated GAPDH at different time points following crotonate addition (Additional file 1: Fig. S3A). GAPDH crotonylation could be clearly detected by day 3, which continued to rise with further treatment. In line with these results, the decrease in the glycolytic rate of these cells began to decrease at day 2 and became more obvious by day 3 (Additional file 1: Figs. S3B-C). By day 6, the rate had dropped to a level similar to that seen in endodermal cells. A similar pattern was seen in the glycolytic capacity of the treated cells (Additional file 1: Figs. S3B-C). These results together support the idea that glycolytic enzymes such as GAPDH and ENOA could be crotonylated in cells and that changes in their crotonylation levels may contribute to metabolic shifts during lineage-specific hESC differentiation.

### Crotonylation regulates the enzymatic activity of GAPDH

**Fig. 5:**
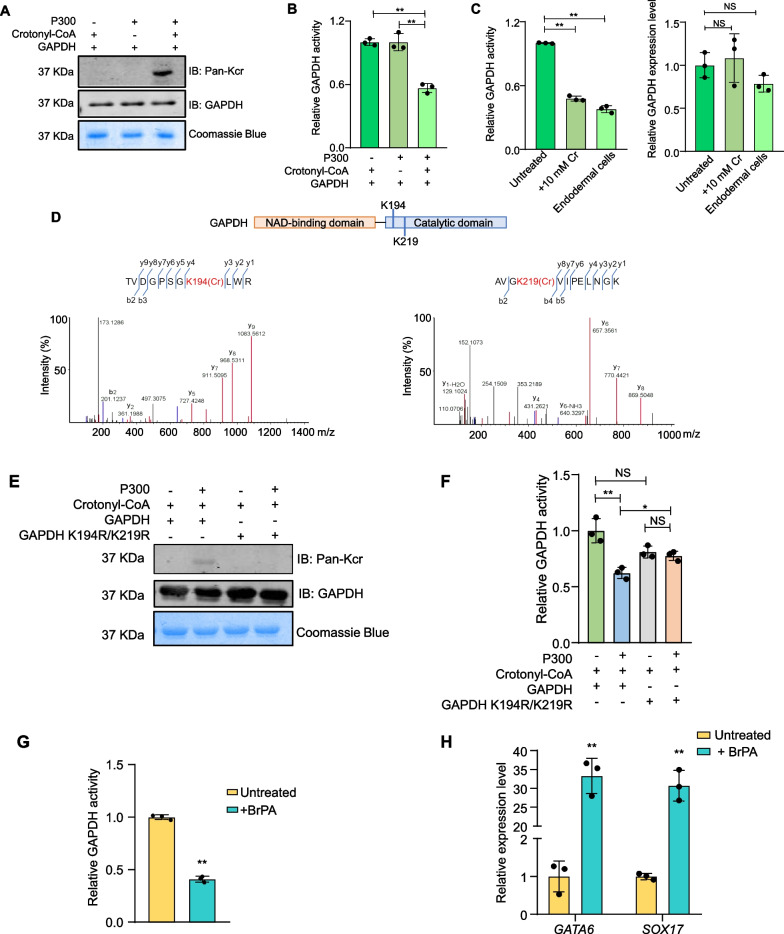
Crotonylation of GAPDH reduces its catalytic activity. **A**, **B** Recombinant GAPDH was incubated with crotonyl-CoA and recombinant p300 and then analyzed by western blotting with the indicated antibodies (**A**) or assessed for GAPDH activity (NAD^+^ conversion rate) (**B**). Coomassie blue staining served as loading control. Error bars represent mean ± S.D. (*n* = 3 independent experiments). Significance was calculated using unpaired *t* test. **p < 0.01. **C** H9-LTR7 GFP hESCs were treated with 10 mM crotonate for 12 days and then harvested to assess the enzymatic activity (NAD^+^ conversion rate) of in vivo crotonylated GAPDH. Untreated cells and endodermal cells derived from the reporter cells served as controls. Error bars represent mean ± S.D. (*n* = 3 independent experiments). Significance was calculated using unpaired *t* test. **p < 0.01. **D** LC–MS/MS identified two potential crotonylation sites on GAPDH. Their respective MS/MS spectra are shown as well. **E**, **F** Recombinant His-tagged wild-type and mutant GAPDH proteins were purified from *E.coli* (BL21) and incubated with crotonyl-CoA and recombinant p300. The reaction products were either resolved by SDS-PAGE and probed with the indicated antibodies (**E**) or assessed for GAPDH activity (NAD^+^ conversion rate) (**F**). Coomassie blue staining served as loading control. Error bars represent mean ± S.D. (*n* = 3 independent experiments). Significance was calculated using unpaired *t* test. *p < 0.05; **p < 0.01. NS, not significant. **G**, **H** H9-LTR7 GFP hESCs were cultured with or without 5 μM 3-BrPA for 16 days before being collected for GAPDH activity assessment (NAD^+^ conversion rate) (**G**) or RT-qPCR analysis of marker gene expression (**H**). Error bars represent mean ± S.D. (*n* = 3 independent experiments). Significance was calculated using unpaired *t* test. **p < 0.01

p300 has been identified as a crotonyltransferase that can catalyze in vitro histone crotonylation in the presence of crotonyl-CoA. We therefore examined whether p300 could similarly modify GAPDH in in vitro crotonylation reactions using recombinant GAPDH and p300. Indeed, in the presence of crotonyl-CoA, robust crotonylation of GAPDH could be detected (Fig. 5A). Next, we assessed the catalytic activity of in vitro modified GAPDH using NAD^+^ as a substrate. Compared to unmodified GAPDH, the ability of GAPDH to convert NAD^+^ significantly decreased in the presence of both p300 and crotonyl-CoA (Fig. 5B). We similarly analyzed ENOA and found that it too could be crotonylated in vitro (Additional file 1: Fig. S3D). Unlike GAPDH, crotonylation appeared to have minimal effect on ENOA activity using D-2-phosphoglycerate as a substrate (Additional file 1: Fig. S3E). Next, we examined the enzymatic activities of in vivo crotonylated GAPDH and ENOA using crotonate-treated reporter hESCs. For comparison, untreated hESCs and derived endodermal cells were included as well. In the case of ENOA, crotonate treatment led to a 50% increase in ENOA activity and differentiated cells exhibited slightly higher activities than untreated hESCs (Additional file 1: Fig. S3F). In contrast, crotonate treatment of hESCs lowered GAPDH activity by ~ 50% in hESCs, to levels similar to that found in the differentiated endodermal cells (Fig. 5C). This decrease is consistent with the observed accumulation of glycolytic metabolites glucose 6-phosphate and fructose 1,6-bisphosphate as well as the reduction of pyruvate levels in crotonate-treated hESCs (Fig. 3C), and points to GAPDH as the major glycolytic enzyme whose activity is tightly regulated by crotonylation during hESC differentiation to the endoderm lineage.

MS analysis identified two potential in vivo crotonylation sites on GAPDH (K194 and K219), both of which are located in the catalytic domain (Fig. 5D). We therefore generated a GAPDH mutant in which both lysines were mutated to arginine (K194R/K219R). As shown in Fig. 5E, mutating the two residues abolished the ability of p300 to crotonylate GAPDH in vitro. Next, we assessed the catalytic activity of wildtype vs. mutant GAPDH (Fig. 5F). In the absence of p300, the double mutant appeared to be slightly reduced in its ability to convert NAD^+^, although this decrease was not statistically significant. Unlike wildtype GAPDH and the single-point mutants (K194 or K219) of GAPDH, whose activities were decreased by crotonylation (Additional file 1: Fig. S3G), the activity of the GAPDH K194R/K219R double mutant remained relatively constant regardless of the presence of p300 (Fig. 5F). These results indicate that the two lysine residues could indeed be crotonylated in vitro and their crotonylation helped regulate GAPDH activity. Given the increased reliance on the TCA cycle rather than glycolysis in crotonate-treated hESCs, we reasoned that modulating GAPDH activity might affect hESC pluripotency. To test this idea, we examined reporter hESCs that had been cultured long term in the presence of the alkylating agent 3-bromo pyruvate (3-BrPA), a glycolytic enzyme inhibitor for which GAPDH is the preferred and primary target [62–64]. Here, 3-BrPA treatment was able to diminish GAPDH activity by > 50% (Fig. 5G). When we measured marker gene expression by RT-qPCR, we found > 30-fold increase in the expression of *GATA6* and *SOX17* in cells treated with 3-BrPA (Fig. 5H). The above findings suggest that modulating GAPDH activity can directly promote endoderm gene expression and provide further evidence that crotonylation of GAPDH may be part of the regulatory process that facilitates the metabolic switch during endodermal differentiation from hESCs.

### Crotonylation of GAPDH is important to endoderm differentiation from hESCs

**Fig. 6:**
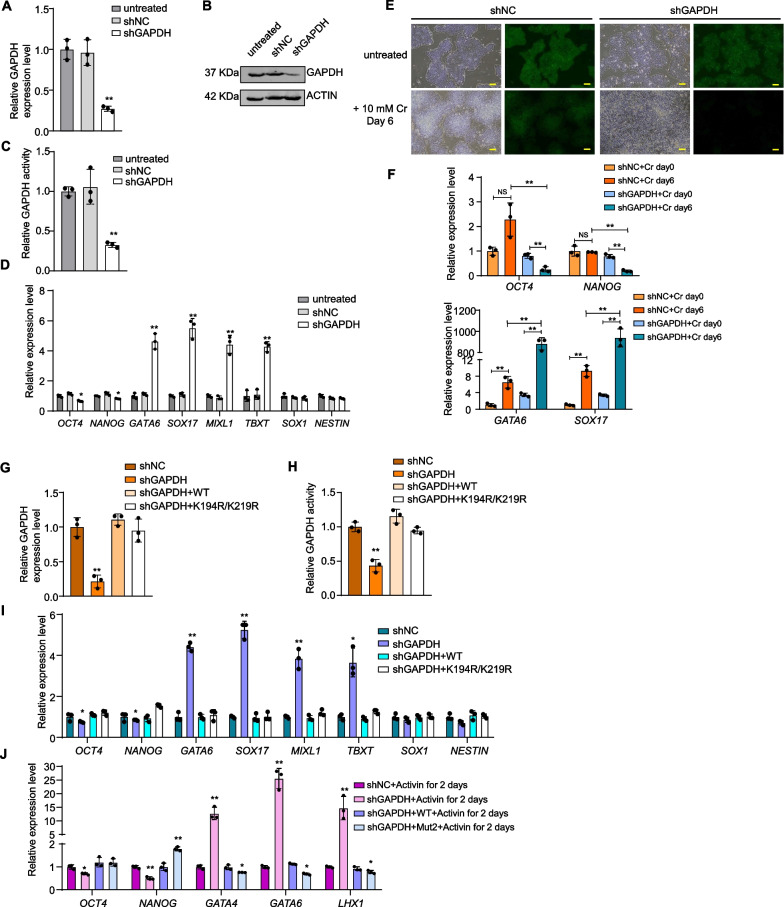
Inhibition of GAPDH expression and/or crotonylation impacts hESC differentiation into the endodermal lineage. **A** shRNA knockdown (KD) of GAPDH (shGAPDH) in H9-LTR7 GFP cells was assessed by RT-qPCR analysis. shNC, non-targeting shRNA. Error bars represent mean ± S.D. (*n* = 3 independent experiments). Significance was calculated using unpaired *t* test. **p < 0.01. **B** KD efficiency was determined by western blotting using the indicated antibodies. **C** GAPDH activity was assessed as described above. Error bars represent mean ± S.D. (*n* = 3 independent experiments). Significance was calculated using unpaired *t* test. **p < 0.01. **D** RT-qPCR analysis of the expression of the indicated marker genes in control and KD cells. *OCT4* and *NANOG*, pluripotency markers. *GATA6* and *SOX17*, endoderm markers. *MIXL1* and *TBXT*, mesoderm markers. *SOX1* and *NESTIN*, ectoderm markers. Error bars represent mean ± S.D. (*n* = 3 independent experiments). Significance was calculated using unpaired *t* test. *p < 0.05; **p < 0.01. **E** GAPDH KD reporter cells were cultured in 10 mM crotonate for 6 days before analysis by microscopy. Untreated cells served as control. Scale bar, 100 μm. **F** RT-qPCR analysis of the indicated marker genes using cells from (**E**). *OCT4* and *NANOG*, pluripotency markers. *GATA6* and *SOX17*, endoderm markers. Error bars represent mean ± S.D. (*n* = 3 independent experiments). Significance was calculated using unpaired *t* test. NS, not significant, **p < 0.01. **G**, **H** GAPDH KD cells stably expressing wildtype or K194R/K219R double mutant GAPDH were examined for GAPDH expression by RT-qPCR (**G**) and GAPDH activity (**H**). Error bars represent mean ± S.D. (*n* = 3 independent experiments). **I** RT-qPCR analysis of the expression of the indicated marker genes in control, KD cells, and GAPDH KD cells stably expressing wildtype or K194R/K219R double mutant GAPDH. Error bars represent mean ± S.D. (*n* = 3 independent experiments). Significance was calculated using unpaired *t* test. *p < 0.05; **p < 0.01. **J** Cells from **G** were cultured in regular endoderm differentiation medium for 2 days before expression analysis of the indicated marker genes. *OCT4* and *NANOG*, pluripotency markers. *GATA4*, *GATA6,* and *LHX1*, endoderm markers. Error bars represent mean ± S.D. (*n* = 3 independent experiments). Significance was calculated using unpaired *t* test. *p < 0.05; **p < 0.01

Although GAPDH is an abundant protein and often used as a loading control, its expression is actually tightly regulated [65] and can vary considerably between different cell types and tissues [66]. When we knocked down (KD) endogenous GAPDH in the reporter hESCs, efficient inhibition of endogenous GAPDH expression could be observed (Fig. 6A–C), which was accompanied by a decrease in GAPDH enzymatic activity in the KD cells. Concurrently, these cells also showed increased endodermal and mesodermal marker gene expression and down-regulated pluripotency marker gene expression (Fig. 6D), adding further evidence that inhibition of GAPDH expression and activity could promote the differentiation of hESCs towards the endodermal lineage. Moreover, the GAPDH KD hESCs appeared more sensitive to crotonate treatment, where highly upregulated endodermal marker gene expression and downregulated pluripotency marker gene expression could be detected by day 6 of treatment (Fig. 6E, F). These results combined support a role of GAPDH in the regulation of hESC lineage-specific differentiation.

Next, we stably expressed wildtype GAPDH and the K194R/K219R double mutant in the KD cells. Exogenous GAPDH was able to restore both expression and activity of GAPDH in the KD cells (Fig. 6G, H). Importantly, when cultured in hESC maintenance medium without crotonate, GAPDH KD-induced increase in endodermal lineage marker expression could be reversed with the rescue expression of both wildtype and double mutant GAPDH (Fig. 6I). These findings are consistent with our BrPA treatment data and provide further evidence that blocking GAPDH activity could promote endodermal cell lineage differentiation. Moreover, when wildtype GAPDH rescued cells were maintained in endodermal differentiation medium, the expression of endodermal (e.g., *GATA4, GATA6,* and *LHX1*) and pluripotency marker genes was similar to that in parental hESCs (Fig. 6J). On the other hand, in KD cells rescued with mutant GAPDH, endodermal marker gene expression decreased slightly and the expression of the pluripotency marker *NANOG* was elevated. These results collectively underline the importance of GAPDH activity regulation and demonstrate that disrupting GAPDH crotonylation could negatively impact endodermal differentiation from hESCs.

## Discussion

The heterogeneity of hESCs has long been a challenge in both developmental studies and clinical applications, where directional differentiation from homogenous cell populations is highly desirable but difficult to achieve. We found that during crotonate-induced hESC differentiation towards the endodermal lineage, cells exhibited highly elevated crotonylation of both histone and non-histone proteins, global gene expression changes, and metabolic pathway switching. Our study highlights the complex and interconnected events that have to occur upon crotonate treatment-induced differentiation of hESCs. The importance of maintaining the equilibrium between small-molecule metabolic intermediates is becoming increasingly appreciated. Often these metabolites can serve as PTM substrates and/or co-factors thereby providing pivotal links between metabolic processes in the cytoplasm and gene expression regulation in the nucleus. In this study, we focused on the role of non-histone protein crotonylation.

Since its discovery, studies of crotonylation have largely focused on histones [27, 32, 34]. Abundant histone crotonylation in the human genome is generally associated with active chromatin [27, 67], and plays an important role in spermatogenesis [28, 39], kidney injury [68], HIV latency [69], and ESC differentiation [32, 33]. In our study, metabolic enzymes appear to be major targets of induced crotonylation as well in hESCs. Interestingly, although the two glycolytic enzymes GAPDH and ENOA could both be crotonylated in vivo and exhibited increased crotonylation upon crotonate treatment, the activity of GAPDH, but not ENOA, was sensitive to levels of crotonylation. When we mutated the two potential crotonylation sites within the GAPDH catalytic domain, the activity of GAPDH became refractory to crotonate treatment. These results are supported by the increase/decrease in glycolytic intermediate metabolites up- and downstream of GAPDH from our metabolomic analysis. Published reports have shown that GAPDH acetylation can enhance its activity in response to glucose [32, 58]. Here, we found that GAPDH crotonylation decreased its catalytic activity, which in turn led to changes during hESC endodermal cell differentiation. Taken together, these observations point to GAPDH as a critical player in regulating hESC pluripotency and promoting endoderm differentiation, and in mediating metabolic switching in response to differentiation-inducing signals. Recently, another group showed that other glycolytic enzymes (e.g., hexokinase 2 and phosphoglycerate kinase 1) could also be crotonylated [70], adding further support to the role of crotonylation in regulating metabolic function in pluripotent stem cells.

The ability to obtain hESCs that are in a more naïve pluripotent state has enabled the investigation of aspects of human development not possible in primed hESCs. EPS cells are thought to have naïve-like cell morphology and in a more pluripotent state. The H9 cells are considered to be in a primed state and therefore more “susceptible” to differentiation induction by exogenous factors. Given that the various interconvertible pluripotency states, we found EPS cells responded to crotonate treatment differently from primed hESCs. Crotonate-treated hESCs resemble endodermal cells in terms of transcriptomic and metabolomic profiles, suggesting that elevating protein crotonylation inside cells may be employed in in vitro differentiation protocols using hESCs of different pluripotent states. This PTM manipulation may be done in conjunction with glycolytic inhibitors, which can further alter the activities of glycolytic enzymes and drive hESCs towards metabolic pathways conducive to germ layer-specific differentiation. Further studies in these areas promise to uncover additional parameters for establishing such protocols and prove beneficial to clinical applications that require pluripotent stem cells.

## Conclusions

Crotonylation of GAPDH decreased its enzymatic activity thereby leading to reduced glycolysis during endodermal differentiation from hESCs.

## Supplementary Information

**Additional file 1**. **Figure S1** (A, B) H9-LTR7 GFP reporter hESCs were cultured first in LCDM medium for 15 passages to derive EPS cells that were immunostained with antibodies against NANOG (A) or OCT4 (B). Primed reporter hESCs served as controls. Scale bars, 100 μm. (C, D) Primed H9-LTR7 GFP reporter hESCs were differentiated as shown. (E, F) The differentiated endodermal cells (E) and ectodermal cells (F) were analyzed by microscopy. (G, H) RT-qPCR assays for lineage marker expression in endodermal cells (G) and ectodermal cells (H) were shown. *OCT4*, *NANOG* and *SOX2* pluripotency markers. *GATA4*, *GATA6* and *SOX17*, endoderm markers. *SOX1*, *NESTIN* and *PAX6*, ectoderm markers. Primed reporter hESCs served as controls. Scale bars, 100 μm. Error bars represent mean ± S.D. (*n* = 3 independent experiments). **Figure S2** (A) The mouse embryonic stem cell line E14 was cultured in the presence of crotonate at the indicated concentrations for 24 h. Whole-cell lysates were probed with the indicated antibodies. (B–F) E14 cells treated for 3 days with the indicated concentrations of acetate or crotonate were examined under microscopes (scale bars of 100 μm) (B). The percentages of naïve and differentiated clones were quantitated and graphed (200 clones/group) for cells treated with acetate (C) or crotonate (D). RT-qPCR analysis of the indicated marker genes was carried out using cells treated with acetate (E) or crotonate (F). Oct4 and Nanog, pluripotency markers. Gata6 and Sox17, endoderm markers. Mixl1 and Cd34, mesoderm markersFull. Sox1 and Nestin, ectoderm markers. Error bars represent mean ± S.D. (*n* = 3 independent experiments). **Figure S3** (A) H9-LTR7 GFP reporter hESCs and hESCs treated with crotonate for 1–6 days were immunoprecipitated (IP) with anti-GAPDH antibody. The immuoprecipitates were then western blotted as indicated. IgG served as a negative control. (B) Extracellular acidification rate (ECAR) analysis of endodermal cells and hESCs treated with 10 mM crotonate for different time points (day 0, day 1, day 2, day 3, day 6, day 12). Glucose (10 mM), oligomycin (1 mM), and 2-deoxyglucose (2-DG, 50 mM) were used. (C) Glycolytic rates and glycolytic capacity for endodermal cells and hESCs treated with 10 mM crotonate for 0 day, 1 day, 2 days, 3 days, 6 days and 12 days. Error bars represent mean ± S.D. (*n* = 3 independent experiments). Significance was calculated using unpaired *t* test. *p < 0.05; **p < 0.01. (D, E) Recombinant ENOA was incubated with crotonyl-CoA and recombinant p300 and analyzed by western blotting with the indicated antibodies (D) or assessed for ENOA activity (E). Coomassie blue staining served as loading control. Error bars represent mean ± S.D. (*n* = 3 independent experiments). (F) H9-LTR7 GFP hESCs were treated with 10 mM crotonate for 12 days and then harvested to assess the activity of in vivo crotonylated ENOA. Untreated cells and endodermal cells derived from the reporter cells served as controls. Error bars represent mean ± S.D. (*n* = 3 independent experiments). (G) Recombinant His-tagged wild-type, single and double point mutant GAPDH proteins were purified from *E.coli* (BL21), incubated with crotonyl-CoA and recombinant p300 and assessed for GAPDH activity. NS, not significant. **p < 0.01.

**Additional file 2. Table S1.** Up-regulated genes in crotonate treated H9-LTR7 GFP cells; **Table S2.** Down-regulated genes in crotonate treated H9-LTR7 GFP cells; **Table S3.** Up-regulated genes in crotonate treated hEPS cells; **Table S4.** Down-regulated genes in crotonate treated hEPS cells; **Table S5.** Large-scale analysis of lysine crotonylated proteins in crotonate treated pluripotent stem cells; **Table S6.** Gene ontology analysis of crotonylated proteins in response to crotonate treatment that were identified by LC/MS-MS; **Table S7.** Quantitative PCR primers used in this study.

## Data Availability

All proteomics data from this study were uploaded to the ProteomeXchange Consortium via the PRIDE repository with the identifier PXD027007. The RNA transcriptomic sequencing data discussed in this publication have been deposited in NCBI's Gene Expression Omnibus and are accessible through GEO Series accession number GSE214619.

## Abbreviations

ESCs: Embryonic stem cells
hESCs: Human embryonic stem cells
PTMs: Post-translational modifications
Ac-CoA: Acetyl-CoA
EPS: Extended pluripotent stem
TCA: Tricarboxylic acid
ECAR: Extracellular acidification rate
3-BrPA: 3-Bromo pyruvate
GAPDH: Glyceraldehyde-3-phosphate dehydrogenase
ENOA: Enolase a

## References

[1] Evans MJ, Kaufman MH (1981). Establishment in culture of pluripotential cells from mouse embryos. Nature.

[2] Martin GR (1981). Isolation of a pluripotent cell line from early mouse embryos cultured in medium conditioned by teratocarcinoma stem cells. Proc Natl Acad Sci U S A.

[3] Thomson JA, Itskovitz-Eldor J, Shapiro SS, Waknitz MA, Swiergiel JJ, Marshall VS, Jones JM (1998). Embryonic stem cell lines derived from human blastocysts. Science.

[4] Varum S, Rodrigues AS, Moura MB, Momcilovic O, Easley CA, Ramalho-Santos J, Van Houten B, Schatten G (2011). Energy metabolism in human pluripotent stem cells and their differentiated counterparts. PLoS ONE.

[5] Zhou WY, Choi M, Margineantu D, Margaretha L, Hesson J, Cavanaugh C, Blau CA, Horwitz MS, Hockenbery D, Ware C, Ruohola-Baker H (2012). Hif1 alpha induced switch from bivalent to exclusively glycolytic metabolism during Esc-to-Episc/Hesc transition. EMBO J.

[6] Zhang J, Khvorostov I, Hong JS, Oktay Y, Vergnes L, Nuebel E, Wahjudi PN, Setoguchi K, Wang G, Do A, Jung HJ, McCaffery JM, Kurland IJ, Reue K, Lee WN, Koehler CM, Teitell MA (2016). Ucp2 regulates energy metabolism and differentiation potential of human pluripotent stem cells. EMBO J.

[7] Perestrelo T, Correia M, Ramalho-Santos J, Wirtz D (2018). Metabolic and mechanical cues regulating pluripotent stem cell fate. Trends Cell Biol.

[8] Chung S, Arrell DK, Faustino RS, Terzic A, Dzeja PP (2010). Glycolytic network restructuring integral to the energetics of embryonic stem cell cardiac differentiation. J Mol Cell Cardiol.

[9] Prigione A, Fauler B, Lurz R, Lehrach H, Adjaye J (2010). The senescence-related mitochondrial/oxidative stress pathway is repressed in human induced pluripotent stem cells. Stem Cells.

[10] Gu W, Gaeta X, Sahakyan A, Chan AB, Hong CS, Kim R, Braas D, Plath K, Lowry WE, Christofk HR (2016). Glycolytic metabolism plays a functional role in regulating human pluripotent stem cell state. Cell Stem Cell.

[11] Cliff TS, Wu T, Boward BR, Yin A, Yin H, Glushka JN, Prestegaard JH, Dalton S (2017). Myc controls human pluripotent stem cell fate decisions through regulation of metabolic flux. Cell Stem Cell.

[12] Zhu S, Li W, Zhou H, Wei W, Ambasudhan R, Lin T, Kim J, Zhang K, Ding S (2010). Reprogramming of human primary somatic cells by Oct4 and chemical compounds. Cell Stem Cell.

[13] Panopoulos AD, Yanes O, Ruiz S, Kida YS, Diep D, Tautenhahn R, Herrerias A, Batchelder EM, Plongthongkum N, Lutz M, Berggren WT, Zhang K, Evans RM, Siuzdak G, Belmonte JCI (2012). The metabolome of induced pluripotent stem cells reveals metabolic changes occurring in somatic cell reprogramming. Cell Res.

[14] Folmes CDL, Arrell DK, Zlatkovic-Lindor J, Martinez-Fernandez A, Perez-Terzic C, Nelson TJ, Terzic A (2013). Metabolome and metaboproteome remodeling in nuclear reprogramming. Cell Cycle.

[15] Folmes CDL, Martinez-Fernandez A, Faustino RS, Yamada S, Perez-Terzic C, Nelson TJ, Terzic A (2013). Nuclear reprogramming with C-Myc potentiates glycolytic capacity of derived induced pluripotent stem cells. J Cardiovasc Transl Res.

[16] Wellen KE, Hatzivassiliou G, Sachdeva UM, Bui TV, Cross JR, Thompson CB (2009). Atp-citrate lyase links cellular metabolism to histone acetylation. Science.

[17] Zhang J, Nuebel E, Daley GQ, Koehler CM, Teitell MA (2012). Metabolic regulation in pluripotent stem cells during reprogramming and self-renewal. Cell Stem Cell.

[18] Shiraki N, Shiraki Y, Tsuyama T, Obata F, Miura M, Nagae G, Aburatani H, Kume K, Endo F, Kume S (2014). Methionine metabolism regulates maintenance and differentiation of human pluripotent stem cells. Cell Metab.

[19] Carey BW, Finley LW, Cross JR, Allis CD, Thompson CB (2015). Intracellular alpha-ketoglutarate maintains the pluripotency of embryonic stem cells. Nature.

[20] Moussaieff A, Rouleau M, Kitsberg D, Cohen M, Levy G, Barasch D, Nemirovski A, Shen-Orr S, Laevsky I, Amit M, Bomze D, Elena-Herrmann B, Scherf T, Nissim-Rafinia M, Kempa S, Itskovitz-Eldor J, Meshorer E, Aberdam D, Nahmias Y (2015). Glycolysis-mediated changes in acetyl-coa and histone acetylation control the early differentiation of embryonic stem cells. Cell Metab.

[21] Ryall JG, Cliff T, Dalton S, Sartorelli V (2015). Metabolic reprogramming of stem cell epigenetics. Cell Stem Cell.

[22] Ryall JG, Dell'Orso S, Derfoul A, Juan A, Zare H, Feng XS, Clermont D, Koulnis M, Gutierrez-Cruz G, Fulco M, Sartorelli V (2015). The NAD(+)-dependent SIRT1 deacetylase translates a metabolic switch into regulatory epigenetics in skeletal muscle stem cells. Cell Stem Cell.

[23] Chi P, Allis CD, Wang GG (2010). Covalent histone modifications-miswritten, misinterpreted and mis-erased in human cancers. Nat Rev Cancer.

[24] Tessarz P, Kouzarides T (2014). Histone core modifications regulating nucleosome structure and dynamics. Nat Rev Mol Cell Biol.

[25] Wang LH, Zhang T, Wang L, Cai YP, Zhong XY, He XP, Hu L, Tian SY, Wu M, Hui LJ, Zhang HF, Gao P (2017). Fatty acid synthesis is critical for stem cell pluripotency via promoting mitochondrial fission. EMBO J.

[26] Chakrabarty RP, Chandel NS (2021). Mitochondria as signaling organelles control mammalian stem cell fate. Cell Stem Cell.

[27] Tan MJ, Luo H, Lee S, Jin FL, Yang JS, Montellier E, Buchou T, Cheng ZY, Rousseaux S, Rajagopal N, Lu ZK, Ye Z, Zhu Q, Wysocka J, Ye Y, Khochbin S, Ren B, Zhao YM (2011). Identification of 67 histone marks and histone lysine crotonylation as a new type of histone modification. Cell.

[28] Sabari BR, Tang ZY, Huang H, Yong-Gonzalez V, Molina H, Kong HE, Dai LZ, Shimada M, Cross JR, Zhao YM, Roeder RG, Allis CD (2015). Intracellular crotonyl-coa stimulates transcription through P300-catalyzed histone crotonylation. Mol Cell.

[29] Sabari BR, Zhang D, Allis CD, Zhao YM (2017). Metabolic regulation of gene expression through histone acylations. Nat Rev Mol Cell Biol.

[30] Huang H, Wang DL, Zhao YM (2018). Quantitative crotonylome analysis expands the roles of P300 in the regulation of lysine crotonylation pathway. Proteomics.

[31] Wei W, Liu X, Chen J, Gao S, Lu L, Zhang H, Ding G, Wang Z, Chen Z, Shi T, Li J, Yu J, Wong J (2017). Class I histone deacetylases are major histone decrotonylases: evidence for critical and broad function of histone crotonylation in transcription. Cell Res.

[32] Wei W, Mao A, Tang B, Zeng Q, Gao S, Liu X, Lu L, Li W, Du JX, Li J, Wong J, Liao L (2017). Large-scale identification of protein crotonylation reveals its role in multiple cellular functions. J Proteome Res.

[33] Fang Y, Xu X, Ding J, Yang L, Doan MT, Karmaus PWF, Snyder NW, Zhao Y, Li JL, Li X (2021). Histone crotonylation promotes mesoendodermal commitment of human embryonic stem cells. Cell Stem Cell.

[34] Xu W, Wan J, Zhan J, Li X, He H, Shi Z, Zhang H (2017). Global profiling of crotonylation on non-histone proteins. Cell Res.

[35] Wang JC, Singh M, Sun CB, Besser D, Prigione A, Ivics Z, Hurst LD, Izsvak Z (2016). Isolation and cultivation of naive-like human pluripotent stem cells based on HERVH expression. Nat Protoc.

[36] Yang Y, Liu B, Xu J, Wang J, Wu J, Shi C, Xu Y, Dong J, Wang C, Lai W, Zhu J, Xiong L, Zhu D, Li X, Yang W, Yamauchi T, Sugawara A, Li Z, Sun F, Li X, Li C, He A, Du Y, Wang T, Zhao C, Li H, Chi X, Zhang H, Liu Y, Li C, Duo S, Yin M, Shen H, Belmonte JCI, Deng H (2017). Derivation of pluripotent stem cells with in vivo embryonic and extraembryonic potency. Cell.

[37] Chu LF, Leng N, Zhang J, Hou Z, Mamott D, Vereide DT, Choi J, Kendziorski C, Stewart R, Thomson JA (2016). Single-cell Rna-Seq reveals novel regulators of human embryonic stem cell differentiation to definitive endoderm. Genome Biol.

[38] Nakanishi M, Mitchell RR, Benoit YD, Orlando L, Reid JC, Shimada K, Davidson KC, Shapovalova Z, Collins TJ, Nagy A, Bhatia M (2019). Human pluripotency is initiated and preserved by a unique subset of founder cells. Cell.

[39] Liu SM, Yu HJ, Liu YQ, Liu XH, Zhang Y, Bu C, Yuan S, Chen Z, Xie GJ, Li WJ, Xu B, Yang JG, He L, Jin T, Xiong YD, Sun LY, Liu XH, Han CS, Cheng ZY, Liang J, Shang YF (2017). Chromodomain protein Cdyl acts as a crotonyl-Coa hydratase to regulate histone crotonylation and spermatogenesis. Mol Cell.

[40] Gustafson WG, Feinberg BA, McFarland JT (1986). Energetics of beta-oxidation. reduction potentials of general fatty acyl-CoA dehydrogenase, electron transfer flavoprotein, and fatty acyl-CoA substrates. J Biol Chem.

[41] Lenich AC, Goodman SI (1986). The purification and characterization of glutaryl-coenzyme a dehydrogenase from porcine and human liver. J Biol Chem.

[42] Dwyer TM, Rao KS, Goodman SI, Frerman FE (2000). Proton abstraction reaction, steady-state kinetics, and oxidation-reduction potential of human glutaryl-CoA dehydrogenase. Biochemistry.

[43] Li F, Hinderberger J, Seedorf H, Zhang J, Buckel W, Thauer RK (2008). Coupled ferredoxin and crotonyl coenzyme A (CoA) reduction with nadh catalyzed by the butyryl-CoA dehydrogenase/Etf complex from clostridium kluyveri. J Bacteriol.

[44] Wu L, Qiao Y, Gao J, Deng G, Yu W, Chen G, Li D (2011). Functional characterization of rat glutaryl-CoA dehydrogenase and its comparison with straight-chain acyl-CoA dehydrogenase. Bioorg Med Chem Lett.

[45] Miller TE, Beneyton T, Schwander T, Diehl C, Girault M, McLean R, Chotel T, Claus P, Cortina NS, Baret JC, Erb TJ (2020). Light-powered Co_2_ fixation in a chloroplast mimic with natural and synthetic parts. Science.

[46] Wang JC, Xie GC, Singh M, Ghanbarian AT, Rasko T, Szvetnik A, Cai HQ, Besser D, Prigione A, Fuchs NV, Schumann GG, Chen W, Lorincz MC, Ivics Z, Hurst LD, Izsvak Z (2014). Primate-specific endogenous retrovirus-driven transcription defines naive-like stem cells. Nature.

[47] Nichols J, Smith A (2009). Naive and primed pluripotent states. Cell Stem Cell.

[48] Nakamura T, Okamoto I, Sasaki K, Yabuta Y, Iwatani C, Tsuchiya H, Seita Y, Nakamura S, Yamamoto T, Saitou M (2016). A developmental coordinate of pluripotency among mice, monkeys and humans. Nature.

[49] Gao X, Nowak-Imialek M, Chen X, Chen D, Herrmann D, Ruan D, Chen ACH, Eckersley-Maslin MA, Ahmad S, Lee YL, Kobayashi T, Ryan D, Zhong J, Zhu J, Wu J, Lan G, Petkov S, Yang J, Antunes L, Campos LS, Fu B, Wang S, Yong Y, Wang X, Xue SG, Ge L, Liu Z, Huang Y, Nie T, Li P, Wu D, Pei D, Zhang Y, Lu L, Yang F, Kimber SJ, Reik W, Zou X, Shang Z, Lai L, Surani A, Tam PPL, Ahmed A, Yeung WSB, Teichmann SA, Niemann H, Liu P (2019). Establishment of porcine and human expanded potential stem cells. Nat Cell Biol.

[50] Li R, Zhong C, Yu Y, Liu H, Sakurai M, Yu L, Min Z, Shi L, Wei Y, Takahashi Y, Liao HK, Qiao J, Deng H, Nunez-Delicado E, Rodriguez Esteban C, Wu J, Izpisua Belmonte JC (2019). Generation of blastocyst-like structures from mouse embryonic and adult cell cultures. Cell.

[51] Tam PPL, Khoo PL, Wong N, Tsang TE, Behringer RR (2004). Regionalization of cell fates and cell movement in the endoderm of the mouse gastrula and the impact of loss of Lhx1(Lim1) function. Dev Biol.

[52] Heslop JA, Pournasr B, Liu JT, Duncan SA (2021). Gata6 defines endoderm fate by controlling chromatin accessibility during differentiation of human-induced pluripotent stem cells. Cell Rep.

[53] Meistermann D, Bruneau A, Loubersac S, Reignier A, Firmin J, Francois-Campion V, Kilens S, Lelievre Y, Lammers J, Feyeux M, Hulin P, Nedellec S, Bretin B, Castel G, Allegre N, Covin S, Bihouee A, Soumillon M, Mikkelsen T, Barriere P, Chazaud C, Chappell J, Pasque V, Bourdon J, Freour T, David L (2021). Integrated pseudotime analysis of human pre-implantation embryo single-cell transcriptomes reveals the dynamics of lineage specification. Cell Stem Cell.

[54] Acampora D, Simeone A (1999). The tins lecture. Understanding the roles of Otx1 and Otx2 in the control of brain morphogenesis. Trends Neurosci.

[55] Davenne M, Maconochie MK, Neun R, Pattyn A, Chambon P, Krumlauf R, Rijli FM (1999). Hoxa2 and Hoxb2 control dorsoventral patterns of neuronal development in the rostral hindbrain. Neuron.

[56] Kiernan AE, Cordes R, Kopan R, Gossler A, Gridley T (2005). The Notch ligands Dll1 and Jag2 act synergistically to regulate hair cell development in the mammalian inner ear. Development.

[57] Shi L, Tu BP (2015). Acetyl-CoA and the regulation of metabolism: mechanisms and consequences. Curr Opin Cell Biol.

[58] Zhao S, Xu W, Jiang W, Yu W, Lin Y, Zhang T, Yao J, Zhou L, Zeng Y, Li H, Li Y, Shi J, An W, Hancock SM, He F, Qin L, Chin J, Yang P, Chen X, Lei Q, Xiong Y, Guan KL (2010). Regulation of cellular metabolism by protein lysine acetylation. Science.

[59] Li TT, Liu MX, Feng X, Wang Z, Das I, Xu YP, Zhou X, Sun YP, Guan KL, Xiong Y, Lei QY (2014). Glyceraldehyde-3-phosphate dehydrogenase is activated by lysine 254 acetylation in response to glucose signal. J Biol Chem.

[60] Galvan-Pena S, Carroll RG, Newman C, Hinchy EC, Palsson-McDermott E, Robinson EK, Covarrubias S, Nadin A, James AM, Haneklaus M, Carpenter S, Kelly VP, Murphy MP, Modis LK, O'Neill LA (2019). Malonylation of gapdh is an inflammatory signal in macrophages. Nat Commun.

[61] Hou JY, Cao J, Gao LJ, Zhang FP, Shen J, Zhou L, Shi JY, Feng YL, Yan Z, Wang DP, Cao JM (2021). Upregulation of alpha enolase (Eno1) crotonylation in colorectal cancer and its promoting effect on cancer cell metastasis. Biochem Biophys Res Commun.

[62] Ganapathy-Kanniappan S, Geschwind JFH, Kunjithapatham R, Buijs M, Vossen JA, Tchernyshyov I, Cole RN, Syed LH, Rao PP, Ota S, Vali M (2009). Glyceraldehyde-3-phosphate dehydrogenase (Gapdh) is pyruvylated during 3-bromopyruvate mediated cancer cell death. Anticancer Res.

[63] Tang ZJ, Yuan SQ, Hu YM, Zhang H, Wu WJ, Zeng ZL, Yang J, Yun JP, Xu RH, Huang P (2012). Over-expression of GAPDH in human colorectal carcinoma as a preferred target of 3-bromopyruvate propyl ester. J Bioenerg Biomembr.

[64] Seki SM, Stevenson M, Rosen AM, Arandjelovic S, Gemta L, Bullock TNJ, Gaultier A (2017). Lineage-specific metabolic properties and vulnerabilities of T cells in the demyelinating central nervous system. J Immunol.

[65] Tossounian MA, Zhang B, Gout I (2020). The writers, readers, and erasers in redox regulation of GAPDH. Antioxidants.

[66] Barber RD, Harmer DW, Coleman RA, Clark BJ (2005). GAPDH as a housekeeping gene: analysis of GAPDH mRNA expression in a panel of 72 human tissues. Physiol Genom.

[67] Hawkins RD, Hon GC, Lee LK, Ngo Q, Lister R, Pelizzola M, Edsall LE, Kuan S, Luu Y, Klugman S, Antosiewicz-Bourget J, Ye Z, Espinoza C, Agarwahl S, Shen L, Ruotti V, Wang W, Stewart R, Thomson JA, Ecker JR, Ren B (2010). Distinct epigenomic landscapes of pluripotent and lineage-committed human cells. Cell Stem Cell.

[68] Ruiz-Andres O, Sanchez-Nino MD, Cannata-Ortiz P, Ruiz-Ortega M, Egido J, Ortiz A, Sanz AB (2016). Histone lysine crotonylation during acute kidney injury in mice. Dis Model Mech.

[69] Jiang G, Nguyen D, Archin NM, Yukl SA, Mendez-Lagares G, Tang Y, Elsheikh MM, Thompson GR, Hartigan-O'Connor DJ, Margolis DM, Wong JK, Dandekar S (2018). Hiv latency is reversed by ACSS2-driven histone crotonylation. J Clin Investig.

[70] Lv Y, Bu C, Meng J, Ward C, Volpe G, Hu J, Jiang M, Guo L, Chen J, Esteban MA, Bao X, Cheng Z (2021). Global profiling of the lysine crotonylome in different pluripotent states. Genom Proteom Bioinform.

