# Supplementary material for: Crotonylation of GAPDH regulates human embryonic stem cell endodermal lineage differentiation and metabolic switch
Source: Stem Cell Res Ther. 2023 Apr 3;14:63. doi: 10.1186/s13287-023-03290-y (PMC10071711; doi:10.1186/s13287-023-03290-y)
Supplement: Supplementary file 1 — Additional file 1. Figure S1 (A, B) H9-LTR7 GFP reporter hESCs were cultured first in LCDM medium for 15 passages to derive EPS cells that were immunostained with antibodies against NANOG (A) or OCT4 (B). Primed reporter hESCs served as controls. Scale bars, 100 μm. (C, D) Primed H9-LTR7 GFP reporter hESCs were differentiated as shown. (E, F) The differentiated endodermal cells (E) and ectodermal cells (F) were analyzed by microscopy. (G, H) RT-qPCR assays for lineage marker expression in endodermal cells (G) and ectodermal cells (H) were shown. OCT4, NANOG and SOX2 pluripotency markers. GATA4, GATA6 and SOX17, endoderm markers. SOX1, NESTIN and PAX6, ectoderm markers. Primed reporter hESCs served as controls. Scale bars, 100 μm. Error bars represent mean ± S.D. (n = 3 independent experiments). Figure S2 (A) The mouse embryonic stem cell line E14 was cultured in the presence of crotonate at the indicated concentrations for 24 h. Whole-cell lysates were probed with the indicated antibodies. (B–F) E14 cells treated for 3 days with the indicated concentrations of acetate or crotonate were examined under microscopes (scale bars of 100 μm) (B). The percentages of naïve and differentiated clones were quantitated and graphed (200 clones/group) for cells treated with acetate (C) or crotonate (D). RT-qPCR analysis of the indicated marker genes was carried out using cells treated with acetate (E) or crotonate (F). Oct4 and Nanog, pluripotency markers. Gata6 and Sox17, endoderm markers. Mixl1 and Cd34, mesoderm markersFull. Sox1 and Nestin, ectoderm markers. Error bars represent mean ± S.D. (n = 3 independent experiments). Figure S3 (A) H9-LTR7 GFP reporter hESCs and hESCs treated with crotonate for 1–6 days were immunoprecipitated (IP) with anti-GAPDH antibody. The immuoprecipitates were then western blotted as indicated. IgG served as a negative control. (B) Extracellular acidification rate (ECAR) analysis of endodermal cells and hESCs treated with 10 mM crotonate for differ [file 13287_2023_3290_MOESM1_ESM.pdf]

Supplementary Figure 1A,B

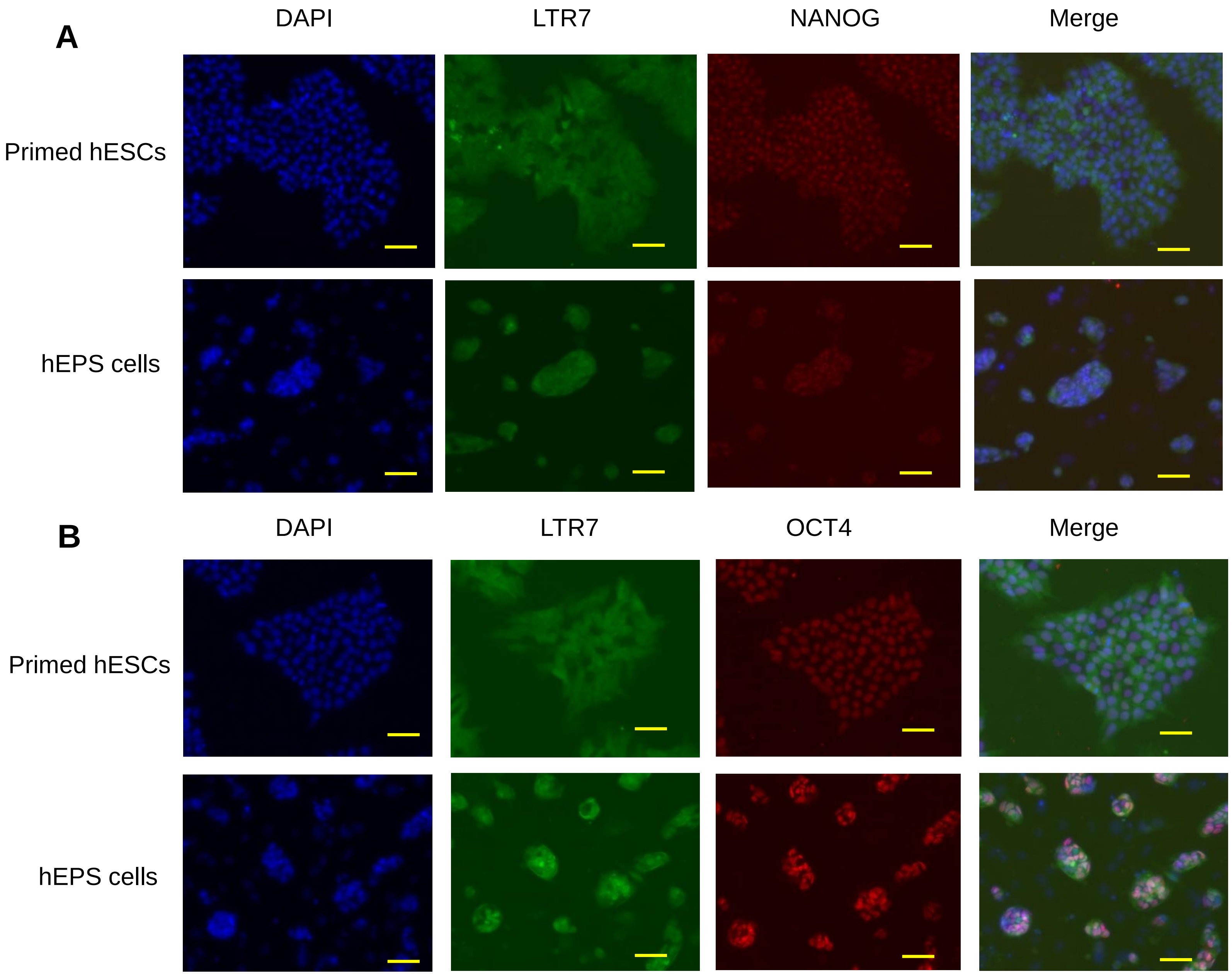

Supplementary Figure 1C-H

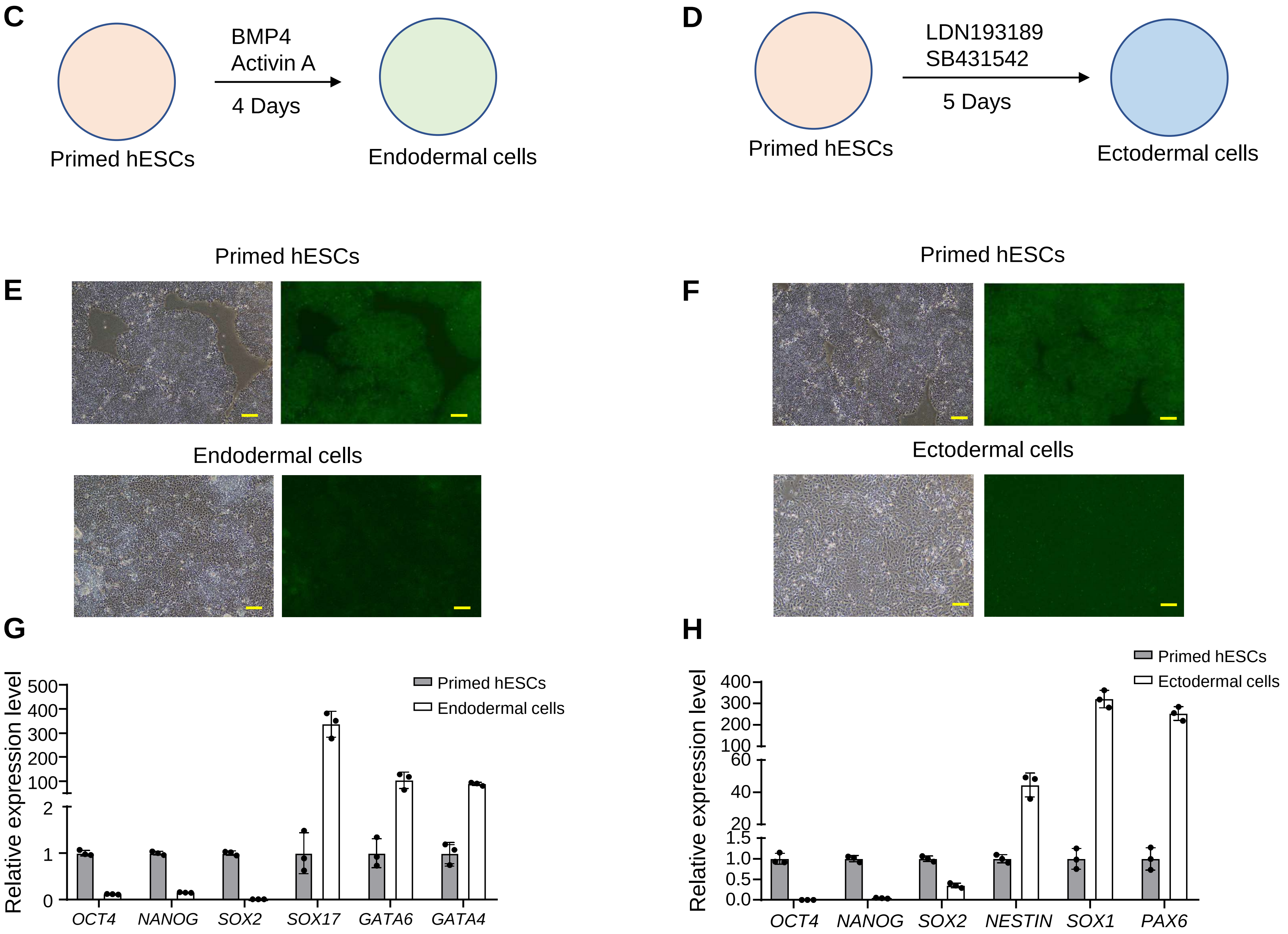

Supplementary Figure 2A-F

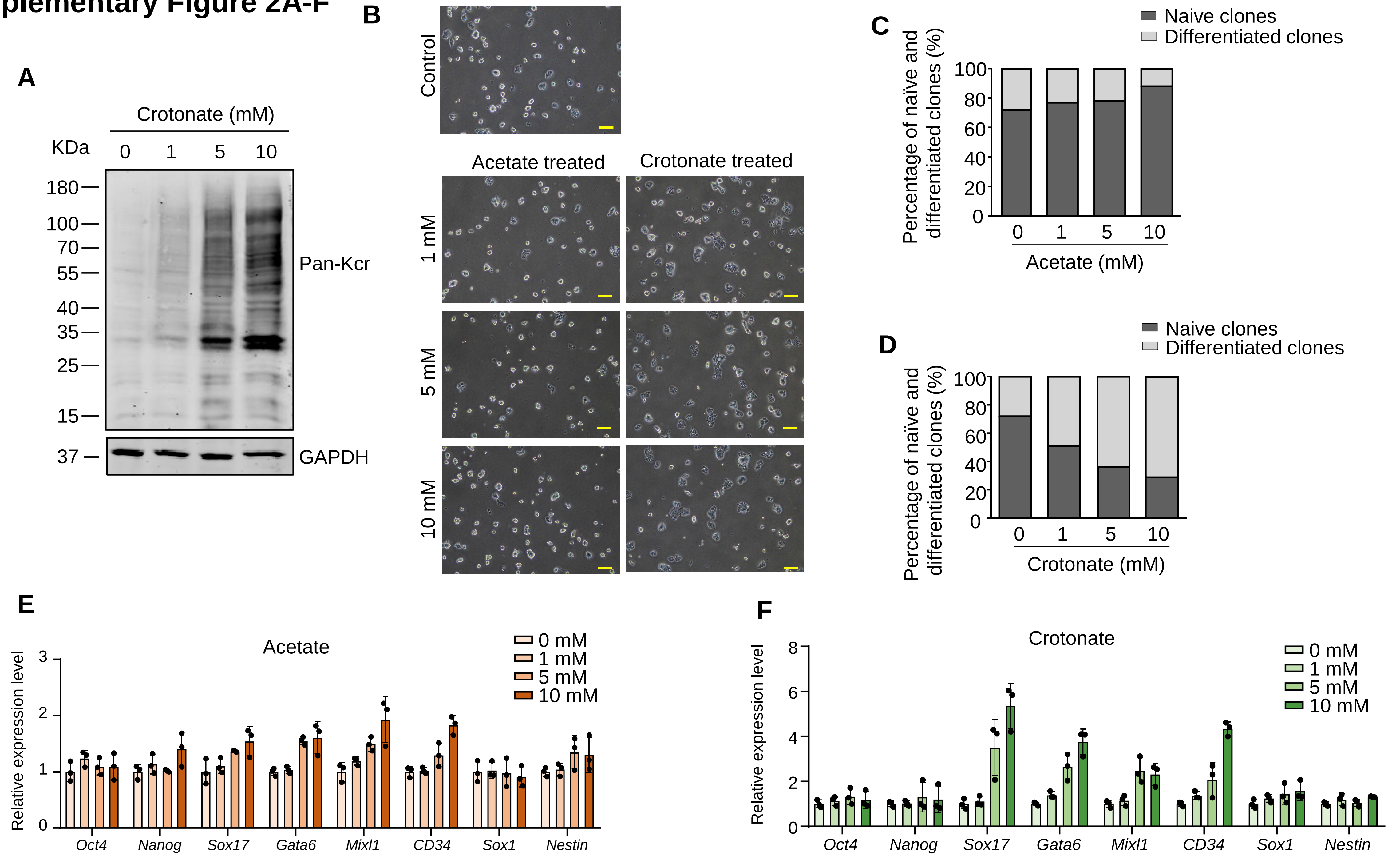

Supplementary Figure 3A-C

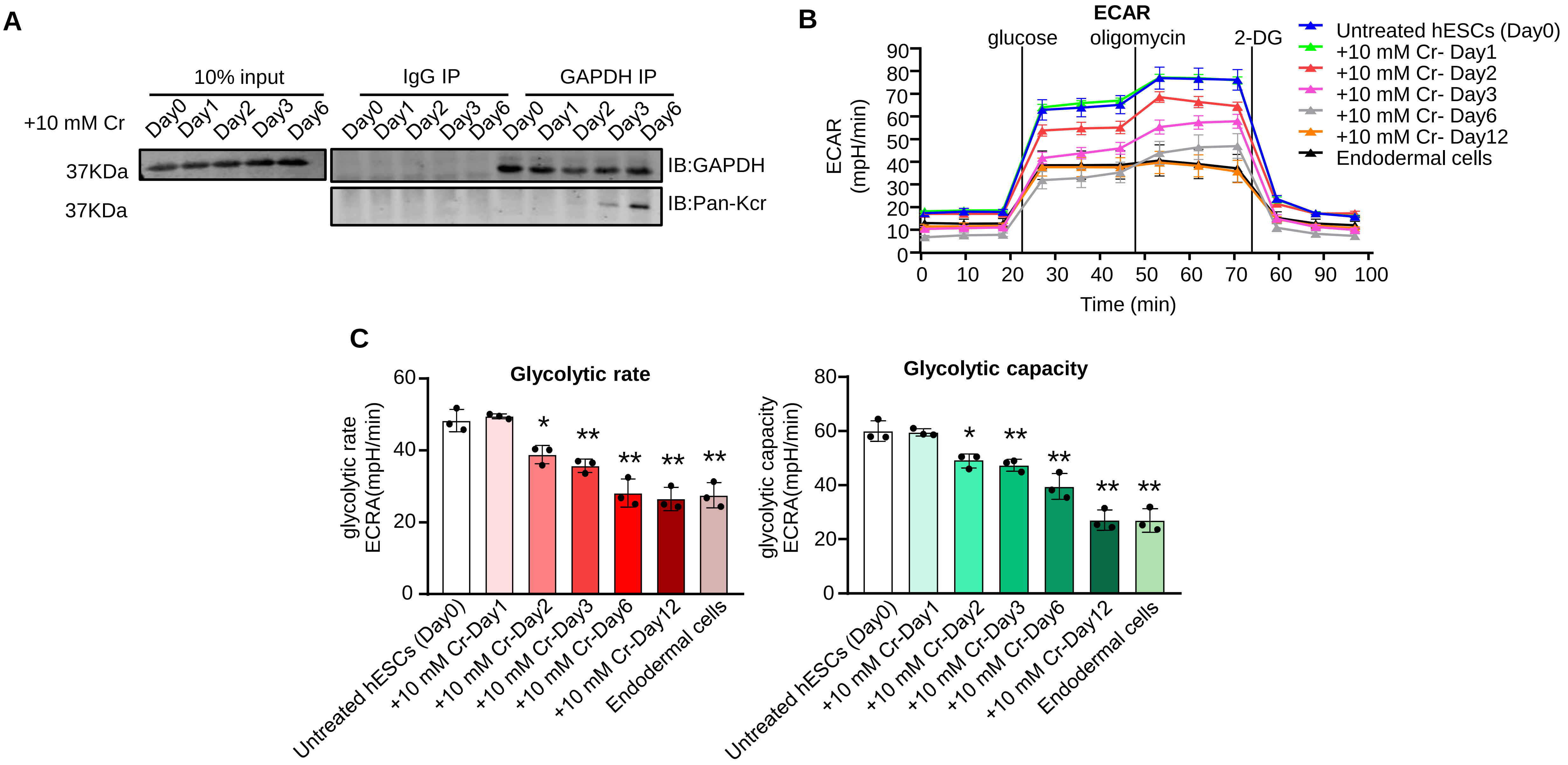

Supplementary Figure 3D-G

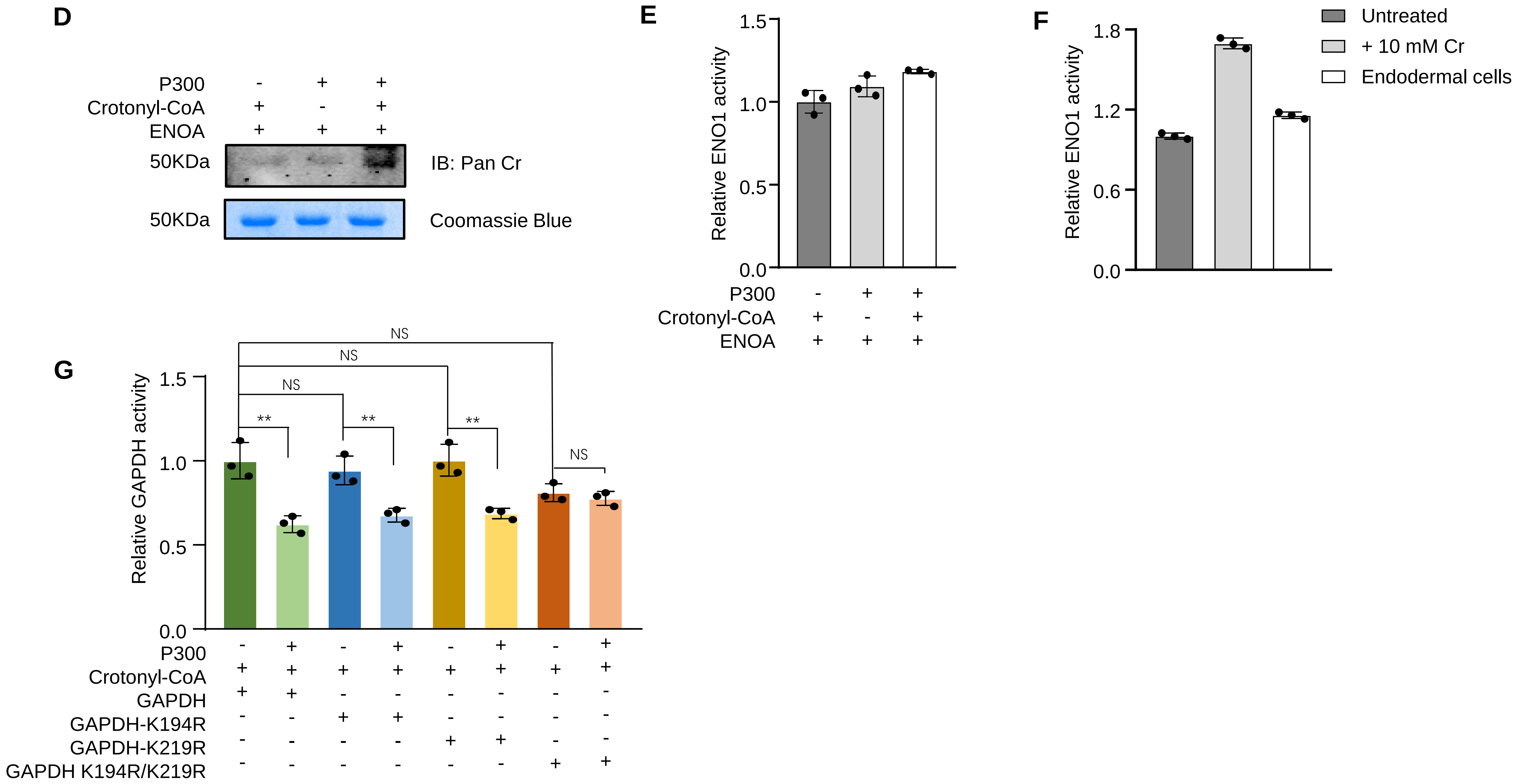

Fig. 2A

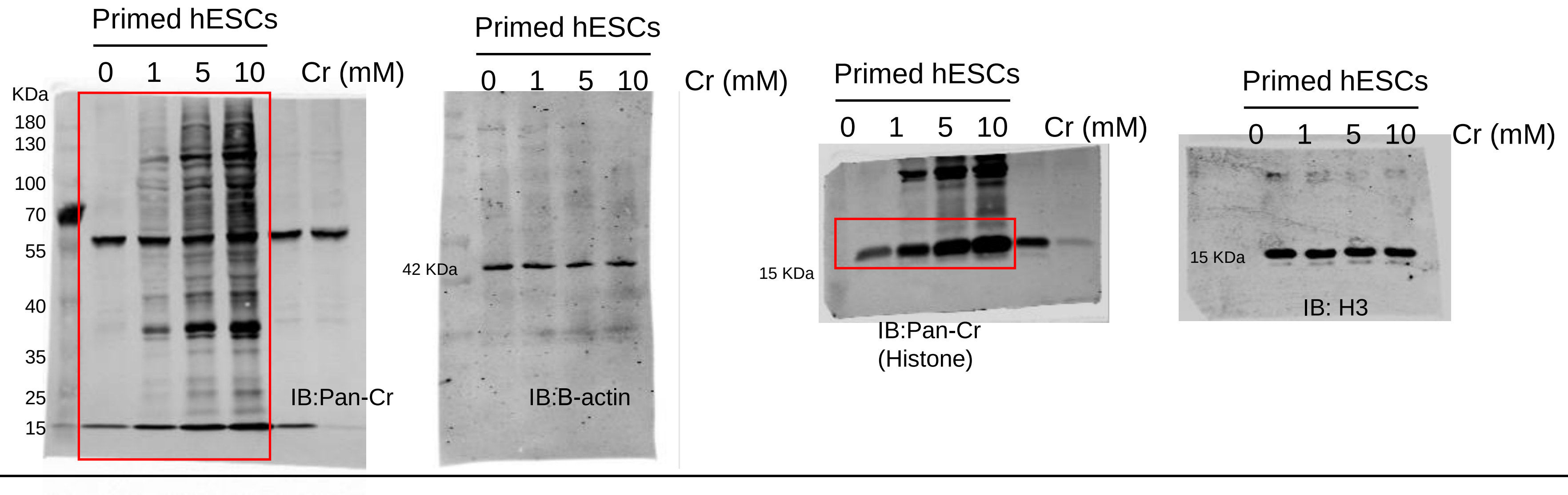
Primed hESCs
0 1 5 10 Cr (mM)
KDa
180
130
100
70
55
40
35
25
15
IB:Pan-Cr
Primed hESCs
0 1 5 10 Cr (mM)
42 KDa
IB:B-actin
Primed hESCs
0 1 5 10 Cr (mM)
15 KDa
IB:Pan-Cr
(Histone)
Primed hESCs
0 1 5 10 Cr (mM)
15 KDa
IB: H3

Fig. 2D

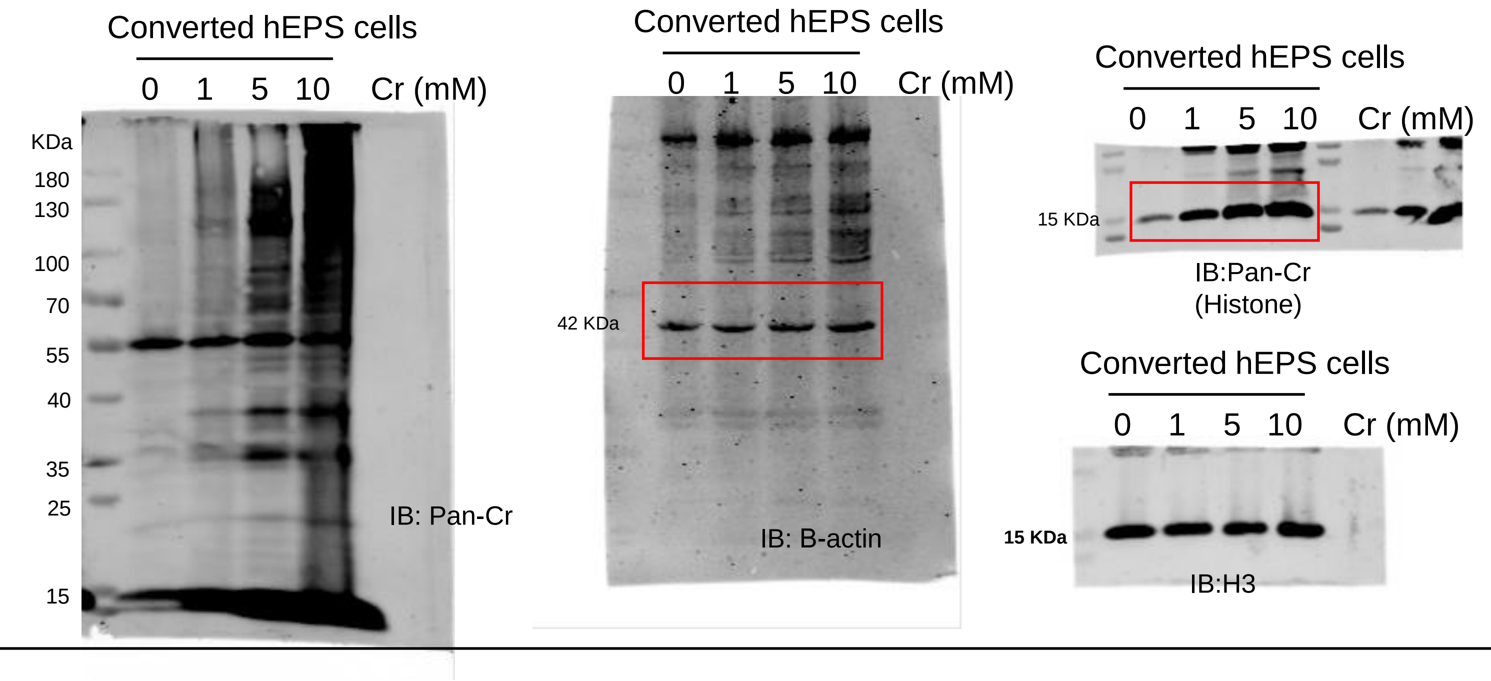
Converted hEPS cells
0 1 5 10 Cr (mM)
KDa
180
130
100
70
55
40
35
25
15
IB: Pan-Cr
Converted hEPS cells
0 1 5 10 Cr (mM)
42 KDa
IB: B-actin
Converted hEPS cells
0 1 5 10 Cr (mM)
15 KDa
IB:Pan-Cr
(Histone)
Converted hEPS cells
0 1 5 10 Cr (mM)
15 KDa
IB:H3

Fig. 2G

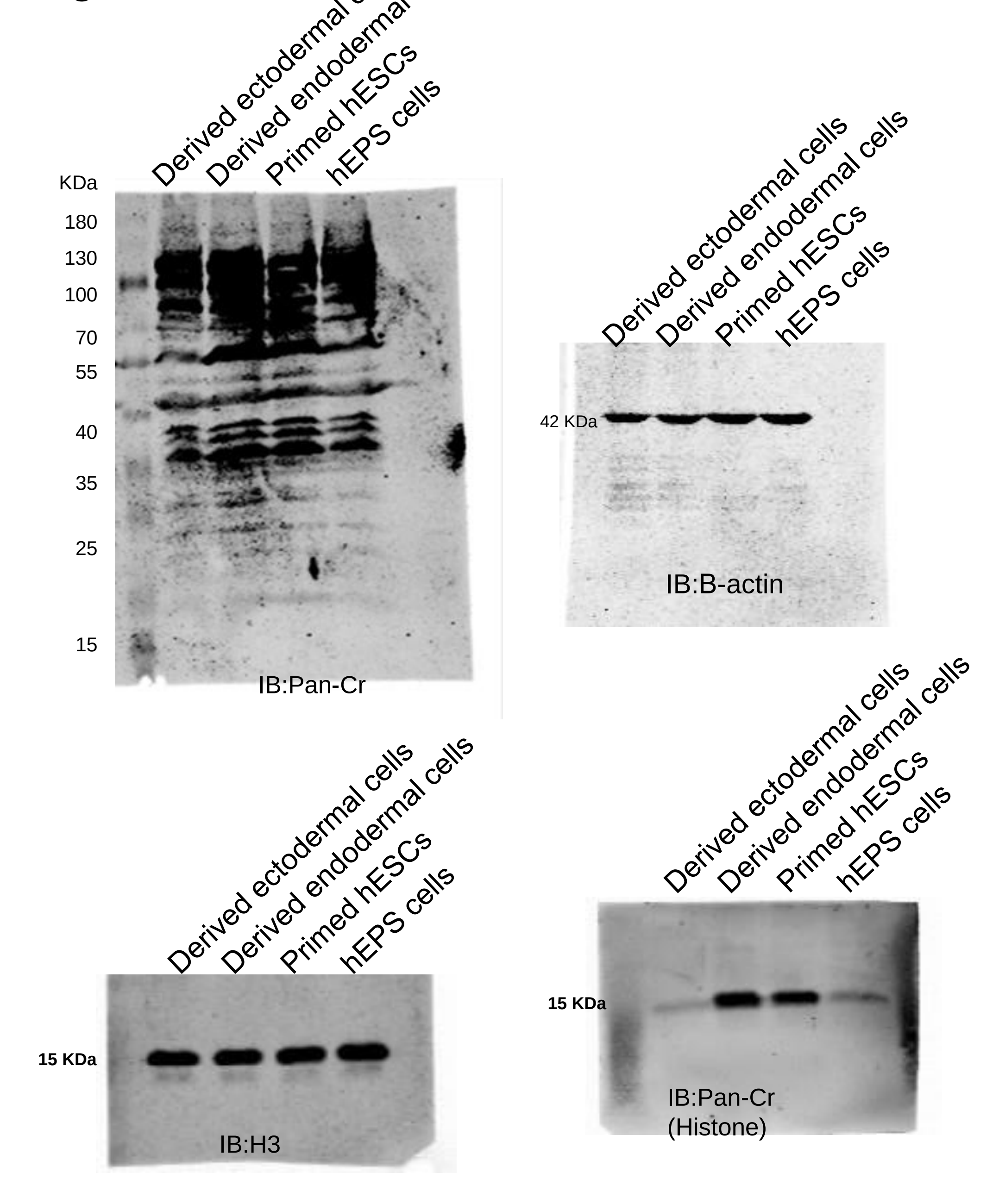
Derived ectodermal cells
Derived endodermal cells
Primed hESCs
hEPS cells
KDa
180
130
100
70
55
40
35
25
15
IB:Pan-Cr
42 KDa
IB:B-actin
15 KDa
IB:H3
IB:Pan-Cr
(Histone)

Fig. 4C

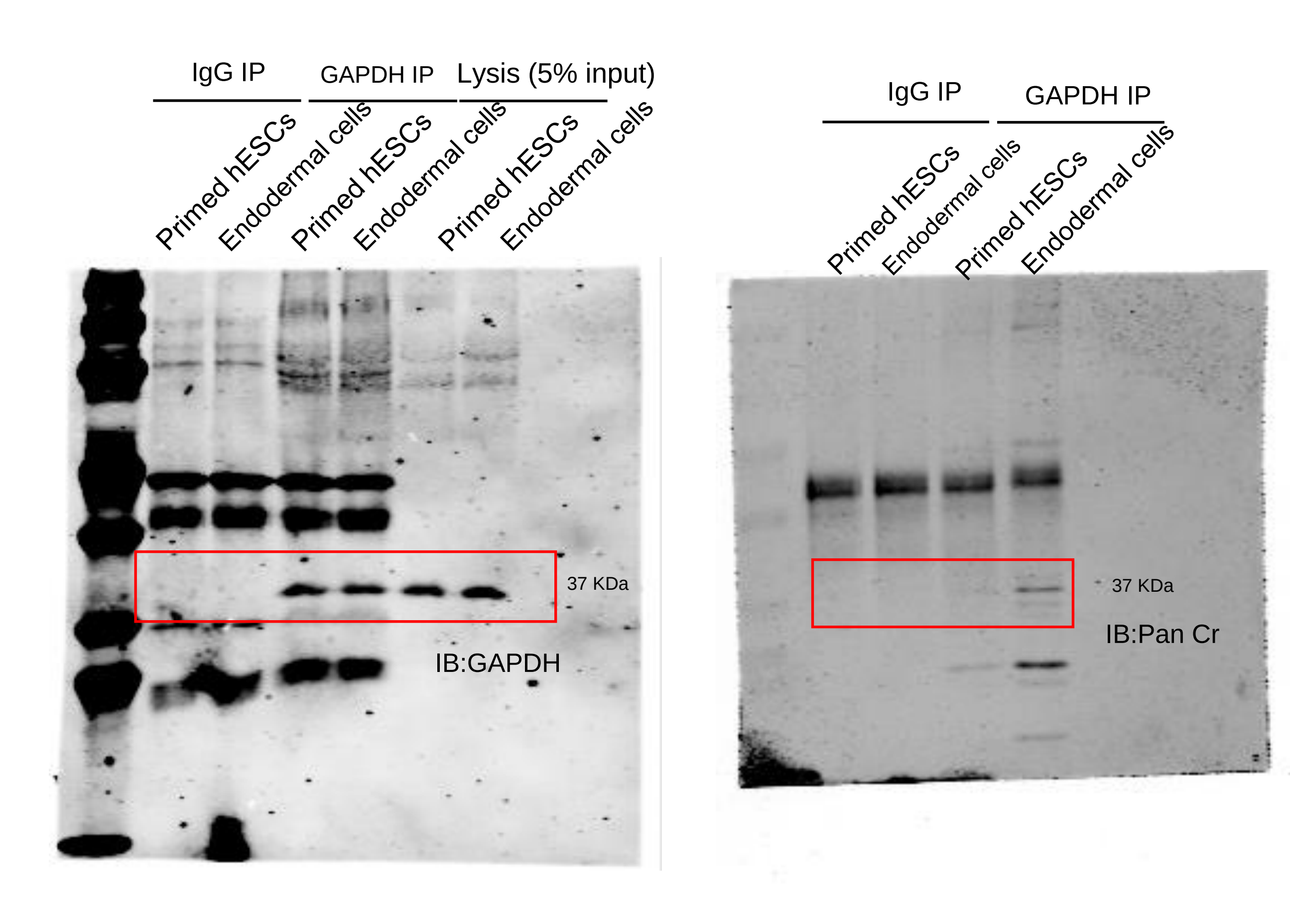
IgG IP
GAPDH IP
Lysis (5% input)
Primed hESCs
Endodermal cells
37 KDa
IB:GAPDH
IB:Pan Cr

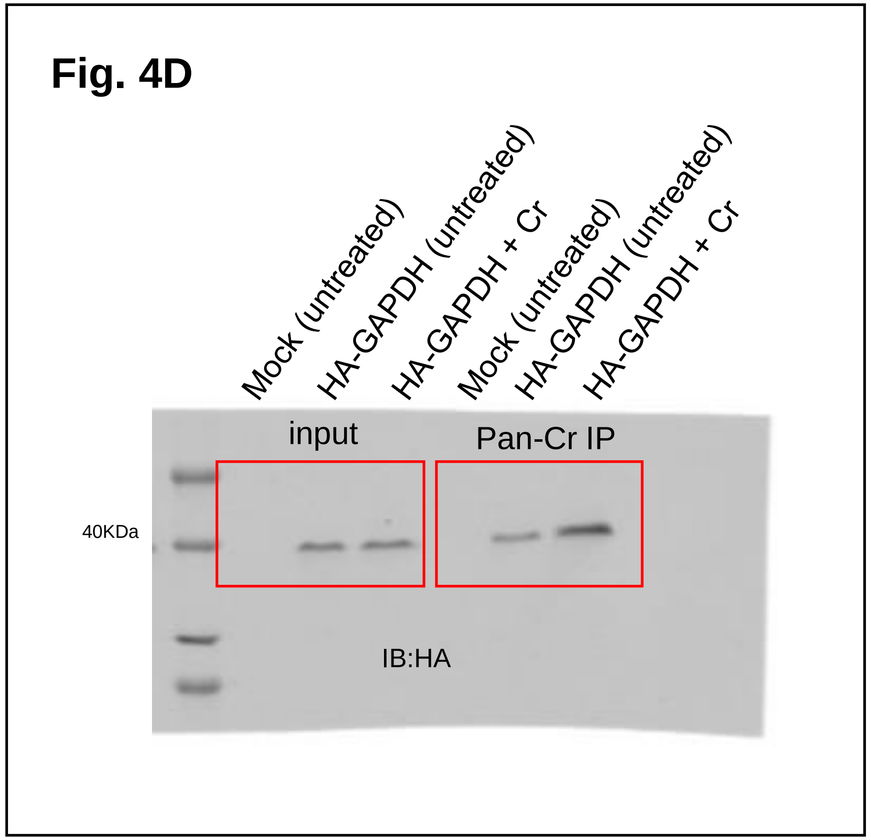
Fig. 4D
Mock (untreated)
HA-GAPDH (untreated)
HA-GAPDH + Cr
Mock (untreated)
HA-GAPDH (untreated)
HA-GAPDH + Cr
input
Pan-Cr IP
40KDa
IB:HA

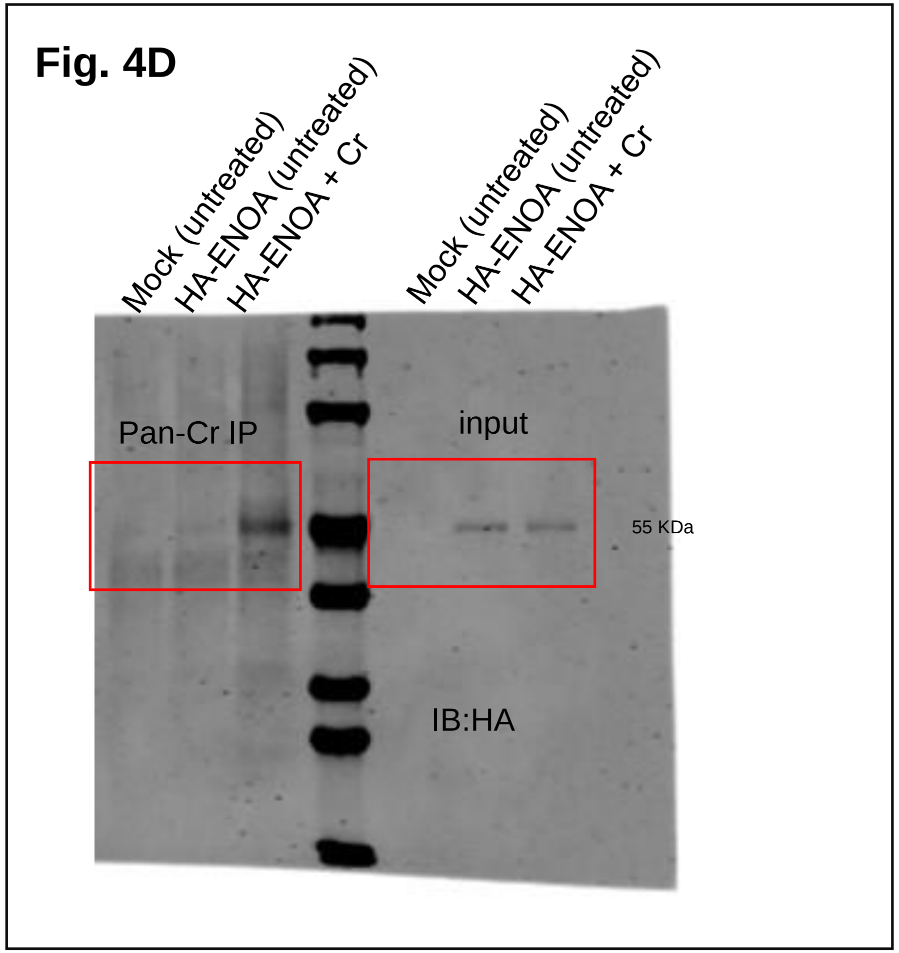
Fig. 4D
Mock (untreated)
HA-ENOA (untreated)
HA-ENOA + Cr
Mock (untreated)
HA-ENOA (untreated)
HA-ENOA + Cr
Pan-Cr IP
input
55 KDa
IB:HA

Fig. 5A

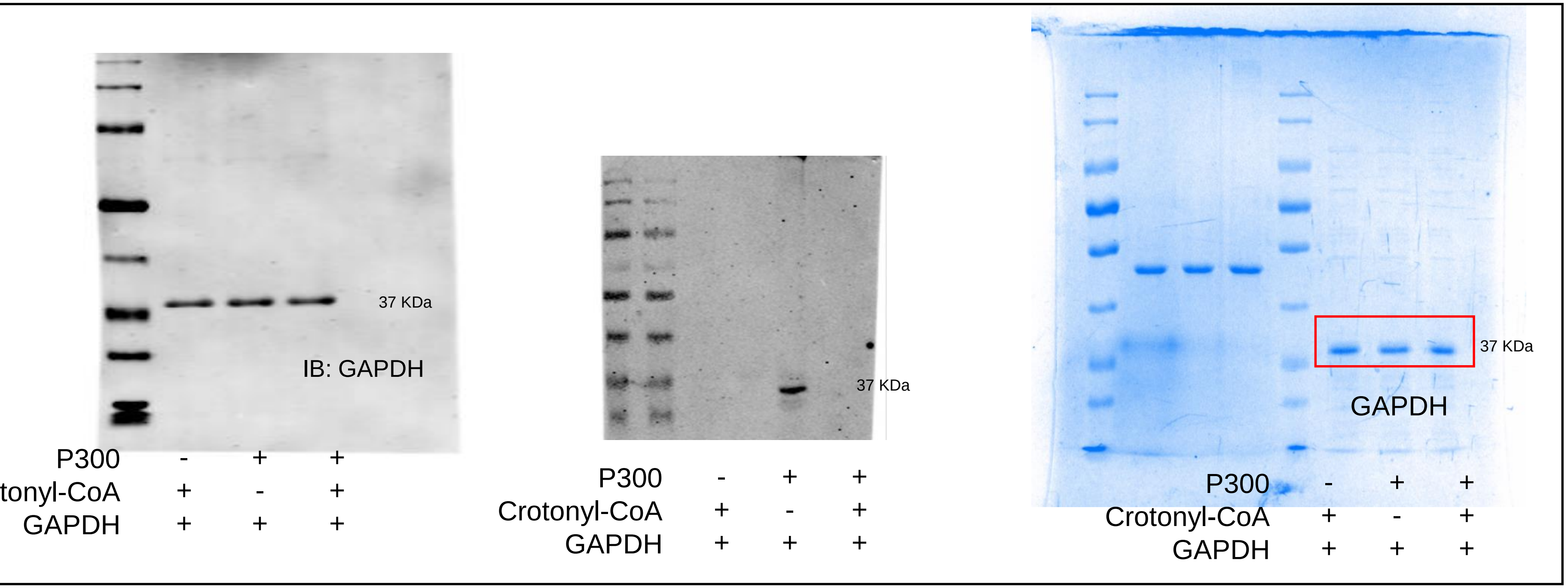
37 KDa
IB: GAPDH
P300 - + +
Crotonyl-CoA + - +
GAPDH + + +
37 KDa
P300 - + +
Crotonyl-CoA + - +
GAPDH + + +
37 KDa
GAPDH
P300 - + +
Crotonyl-CoA + - +
GAPDH + + +

Fig. 5E

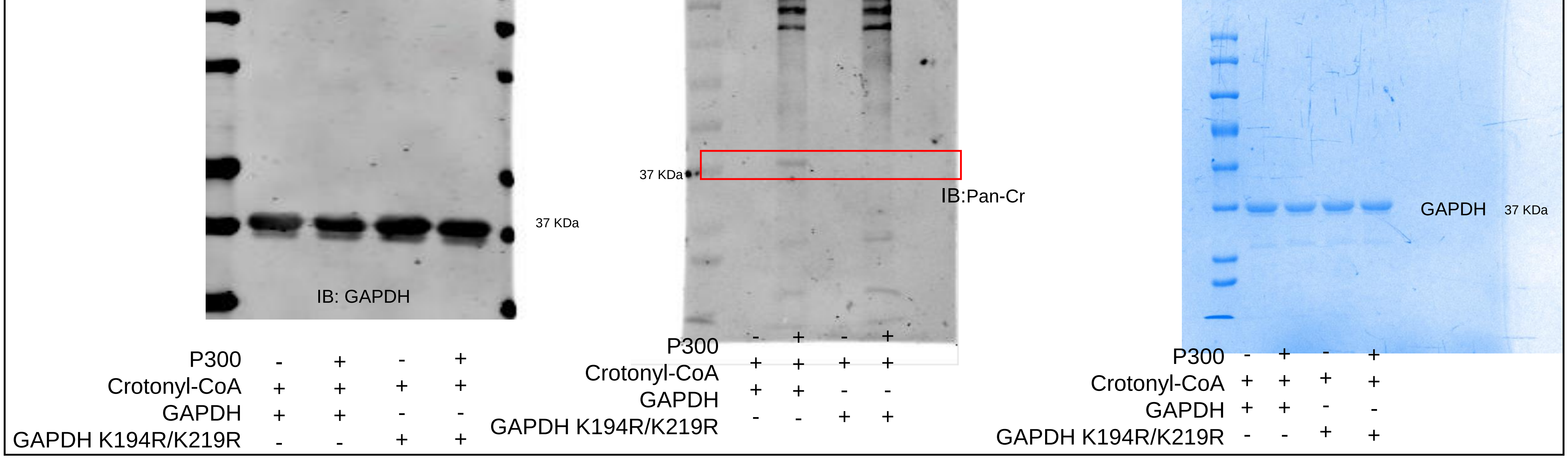
37 KDa
IB: GAPDH
P300 - + - +
Crotonyl-CoA + + + +
GAPDH + + - -
GAPDH K194R/K219R - - + +
37 KDa
IB:Pan-Cr
P300 - + - +
Crotonyl-CoA + + + +
GAPDH + + - -
GAPDH K194R/K219R - - + +
GAPDH
37 KDa
P300 - + - +
Crotonyl-CoA + + + +
GAPDH + + - -
GAPDH K194R/K219R - - + +

Fig. 6B

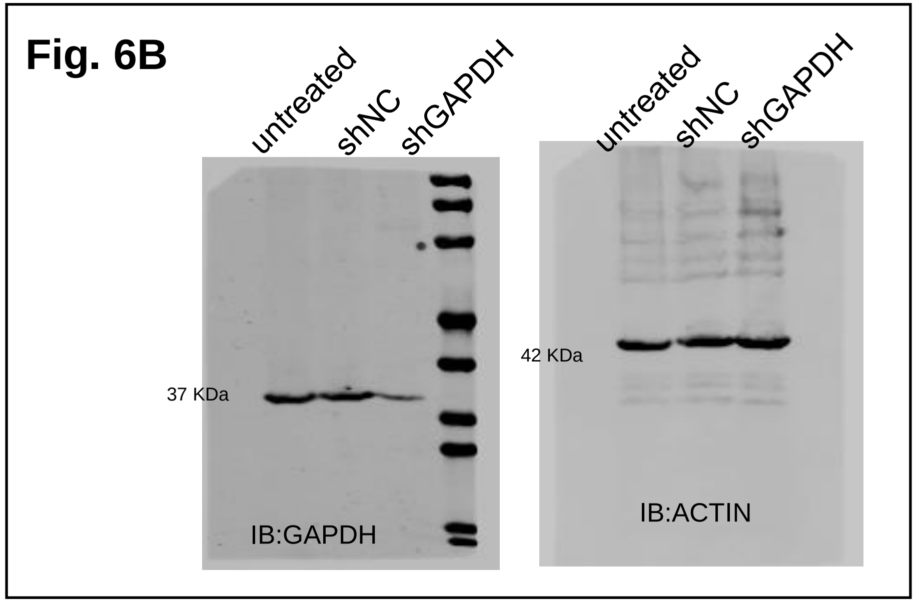

Fig. S2A

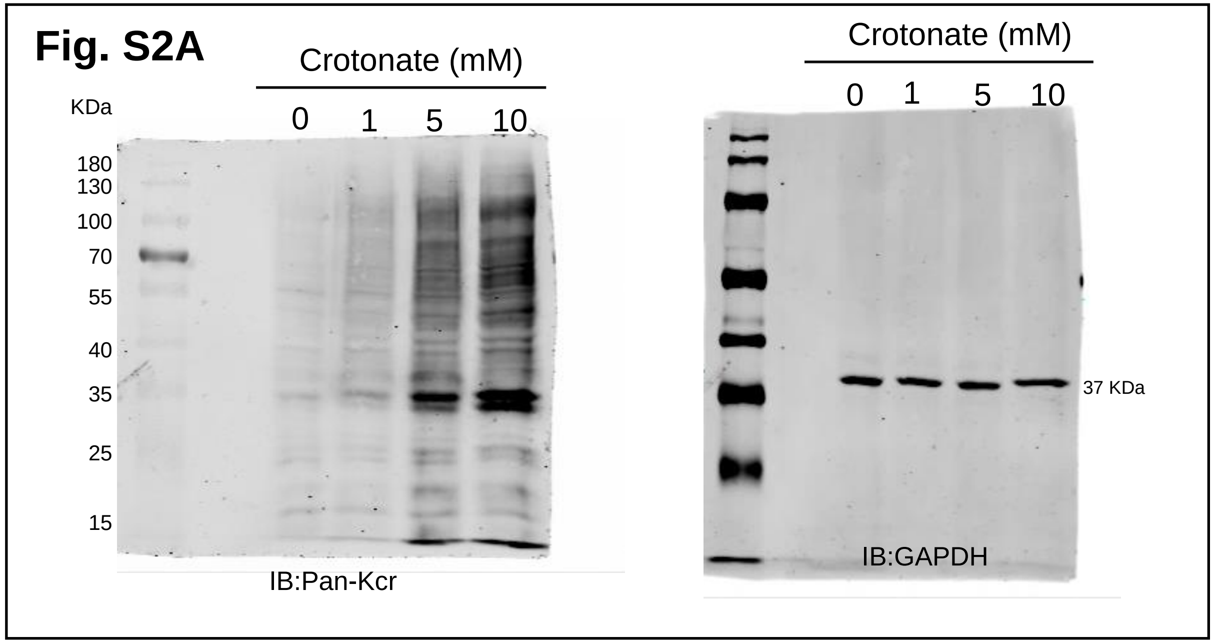

Fig. S3A

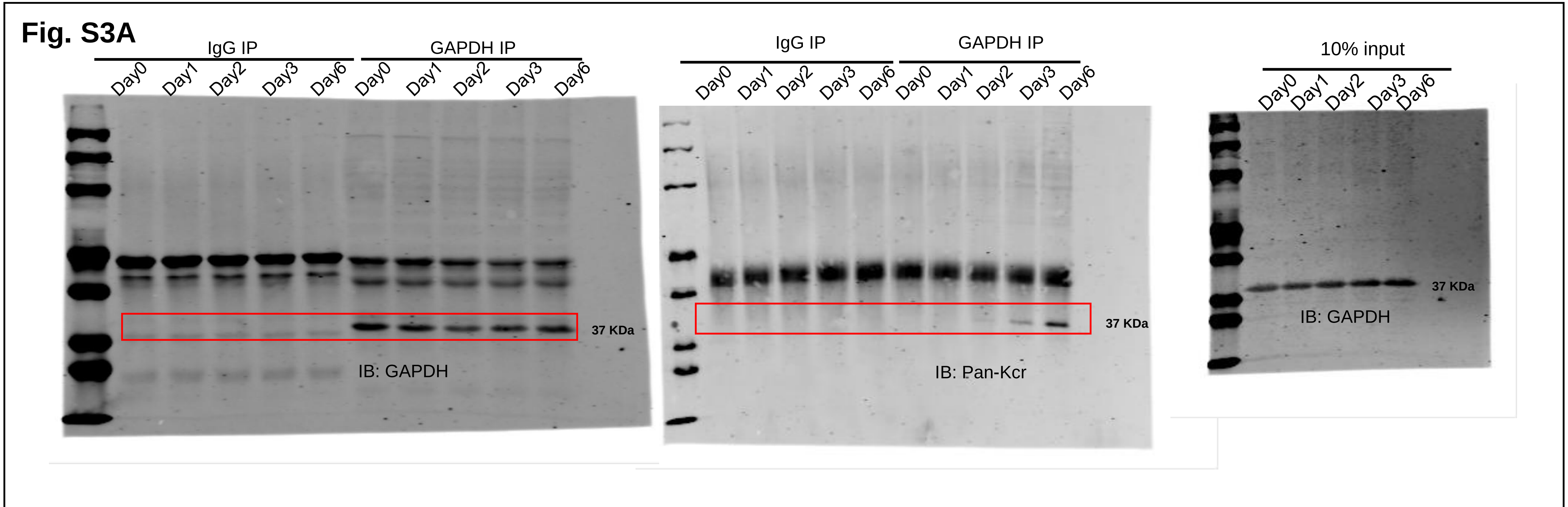

Fig. S3D

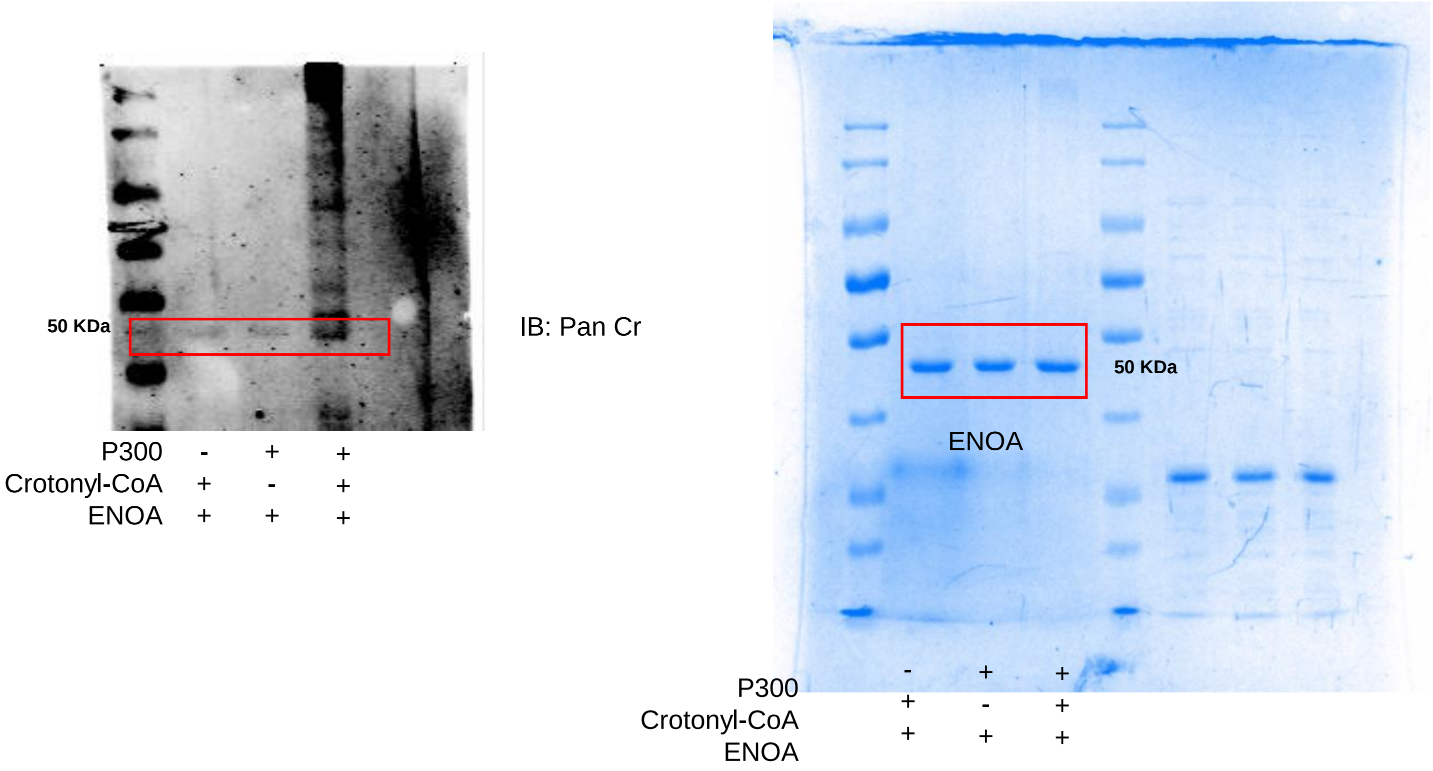
50 KDa
IB: Pan Cr
P300 - + +
Crotonyl-CoA + - +
ENOA + + +
50 KDa
ENOA
P300 - + +
Crotonyl-CoA + - +
ENOA + + +
